# Establishing the content validity of patient-reported outcome measures used in neuro-oncology based on the WHO ICF framework: Part of the RANO-PRO initiative

**DOI:** 10.1093/neuonc/noaf108

**Published:** 2025-04-28

**Authors:** Ogechukwu A Asogwa, Linda Dirven, Tobias Walbert, Terri S Armstrong, David Arons, Martin J van den Bent, Jaishri Blakeley, Marijke B Coomans, Paul D Brown, Helen Bulbeck, Susan M Chang, Corneel Coens, Mark R Gilbert, Robin Grant, Rakesh Jalali, Johan A F Koekkoek, Pankaj Kumar Panda, Danielle Leach, Heather Leeper, Tito Mendoza, Lakshmi Nayak, Kathy Oliver, Jaap C Reijneveld, Emilie Le Rhun, Larry Rubinstein, Jennie W Taylor, Michael Weller, Patrick Y Wen, Martin J B Taphoorn

**Affiliations:** Department of Neurology, Leiden University Medical Center, Leiden, the Netherlands; Department of Neurology, Leiden University Medical Center, Leiden, the Netherlands; Department of Neurology and Neurosurgery, Henry Ford Health System, Wayne State University and Michigan State University, Detroit, Michigan, USA; Neuro-Oncology Branch, Center for Cancer Research, National Cancer Institute, National Institutes of Health, Bethesda, Maryland, USA; National Brain Tumor Society, Newton, Massachusetts, USA; Department of Neurology, Erasmus MC Cancer Institute, Rotterdam, the Netherlands; Department of Neurology, Johns Hopkins University School of Medicine, Baltimore, Maryland, USA; Department of Neurology, Leiden University Medical Center, Leiden, the Netherlands; Department of Radiation Oncology, Mayo Clinic, Rochester, Minnesota, USA; Brainstrust—The Brain Cancer People, Cowes, Isle of Wight, UK; Division of Neuro-Oncology, Department of Neurological Surgery, University of California, San Francisco, California, USA and UCSF Brain Tumor Center, University of California, San Francisco, California, USA; Statistical Department, European Organization for Research and Treatment of Cancer Headquarters, Brussels, Belgium; Neuro-Oncology Branch, Center for Cancer Research, National Cancer Institute, National Institutes of Health, Bethesda, Maryland, USA; Department of Clinical Neurosciences, Royal Infirmary of Edinburgh, Edinburgh, UK; Neuro-Oncology Cancer Management Team, Apollo Proton Cancer Centre, Chennai, India; Department of Neurology, Leiden University Medical Center, Leiden, the Netherlands; Clinical Research Secretariat, Apollo Proton Cancer Centre, Chennai, India; National Brain Tumor Society, Newton, Massachusetts, USA; Department of Neurology and Department of Medicine, Section of Geriatrics and Palliative Medicine, University of Chicago, Chicago, Illinois, USA; Office of Patient-Centered Outcomes Research, Center for Cancer Research, National Cancer Institute, National Institutes of Health, Bethesda, Maryland, USA; Center for Neuro-Oncology, Dana-Farber Cancer Institute, Boston, Massachusetts, USA; International Brain Tumour Alliance, Tadworth, Surrey, UK; Department of Neurology, Stichting Epilepsie Instellingen Nederland (SEIN), Heemstede, the Netherlands; Department of Neurology and Brain Tumor Center Amsterdam, Amsterdam University Medical Centers, Amsterdam, the Netherlands; Department of Neurology, Clinical Neuroscience Center, University Hospital and University of Zurich, Zurich, Switzerland; Department of Neurosurgery, Clinical Neuroscience Center, University Hospital and University of Zurich, Zurich, Switzerland; Biometric Research Program, National Cancer Institute, National Institutes of Health, Bethesda, Maryland, USA; Division of Neuro-Oncology, Department of Neurological Surgery, University of California, San Francisco, California, USA and UCSF Brain Tumor Center, University of California, San Francisco, California, USA; Department of Neurology, Clinical Neuroscience Center, University Hospital and University of Zurich, Zurich, Switzerland; Center for Neuro-Oncology, Dana-Farber Cancer Institute, Boston, Massachusetts, USA; Department of Neurology, Leiden University Medical Center, Leiden, the Netherlands

**Keywords:** brain tumor, content coverage, content validity, patient-reported outcome (PRO) measure, WHO International Classification of Functioning, Disability, and Health (ICF) framework

## Abstract

**Background:**

Instruments to assess patient-reported outcomes (PRO) should generate high-quality evidence. Reliable PRO evidence is essential to policymakers, in conjunction with outcomes such as survival and radiological response, to understand the net clinical benefit of antitumor treatments. This study aimed to establish the content validity of 215 identified PRO measures used in patients with brain tumors.

**Methods:**

A survey (*n* = 148 items) was developed reflecting aspects of the WHO International Classification of Functioning, Disability, and Health (ICF) framework. Patients with brain tumors, their proxies, and healthcare professionals (HCPs) were asked to rate each survey item on relevance. An item was considered a relevant issue if ≥25% of the patients or proxies or ≥50% of the HCPs considered that item to be an issue. Next, all items in the identified PRO measures were linked to ICF and relevant items in the survey, and the percentage of content coverage was calculated.

**Results:**

In total, 114 patients, 71 proxies, and 65 HCPs from different countries completed the survey. Fifty-six of 148 (37.8%) items in the survey were considered relevant. The most important aspects mentioned by both patients and proxies were difficulty concentrating, difficulty remembering, multitasking, and handling stress. Depending on the definition, between 35% and 49% of PRO measures were considered to have sufficient content validity (≥80% coverage).

**Conclusion:**

The content validity was insufficient in more than half of the identified PRO measures, particularly multidimensional measures. Future research should investigate whether different approaches to PRO assessment better meet the needs of all stakeholders.

**Key points**

- Fifty-six ICF items were relevant for patient, proxy, or HCP.
- Patients and proxies reported concentrating, remembering, multitasking, and handling stress as the most relevant items.
- Content validity was insufficient in more than half of the PRO measures.

**Importance of the Study**

PROs are essential in oncology drug development and regulatory review. However, its implementation within regulatory decision-making is hampered by the limited use of appropriate PRO measures, specifically in the brain tumor population. To date, no recommendations are provided on the appropriate tools to assess PRO in the brain tumor populations. Additionally, it is unclear whether PRO measures exhibit good content validity, given that PRO measures are needed to generate high-quality evidence in clinical trials and practice. We assessed the content validity of 215 unique identified PRO measures from brain tumor studies by linking them to the WHO ICF framework. Our findings show that 56/148 (37.8%) ICF items were considered relevant, and the most important aspects mentioned by patients and proxies were difficulty concentrating, remembering, multitasking, and handling stress. Moreover, about 35%-49% of PRO measures have sufficient content validity (≥80% coverage), and content validity was insufficient, particularly in multidimensional measures.

The Response Assessment in Neuro-Oncology—Patient-Reported Outcome (RANO-PRO) working group is an international multidisciplinary collaboration that has been established to provide guidance on the use of PRO measures in clinical trials and routine clinical practice for adults with primary and secondary brain tumors.^1^ Findings from both patient-centered outcome measures (including clinician-reported outcomes, observer-reported outcomes, and PROs) and disease-focused outcome measures (such as survival and clinical or radiological response) are essential to assist physicians and patients in the treatment decision-making process, to inform the research community and policymakers on the net clinical benefit of a tumor-directed treatment, and to assess the financial consequences of a tumor and its treatment, as well as the impact of the disease on the functioning and well-being of the patients’ caregiver. In clinical practice, routine assessment and discussion of PRO results have been shown to improve communication between the patient and the physician, to redirect the focus of the consultation on topics that are important to the patient, to improve the patient's level of health-related quality of life, and to even prolong survival.^2–6^ Moreover, the patient perspective, as assessed with PRO measures, is an essential component of oncology drug development and regulatory review.^7^ However, the implementation of PROs within regulatory decision-making has been hampered by the limited use and availability of appropriate PRO measures. Currently, the drug development community is asking for more patient-centered data in regulatory approval.^8^ Therefore, in both clinical trial and practice settings, relevant and appropriate PRO measures are needed to generate high-quality evidence.

Prior initiatives in oncology focused on guidelines concerning the collection, analysis, interpretation, and reporting of PRO data.^9–14^ The RANO-PRO initiative focuses on providing recommendations for applying appropriate PRO instruments with respect to their content and measurement properties in brain tumor research.^1^ PROs should be well defined and reliable to generate high-quality evidence. Recently, the Fast Track Clinical Outcome Assessment (COA) Group, comprising several RANO-PRO working group members, defined a core set of symptoms and functions that should be assessed in all trials for high-grade glioma patients.^15^ To date, no recommendations have been made on specific measures to assess these constructs. The RANO-PRO working group’s recommendations on the use of specific PRO measures could help improve the quality of PRO evidence derived from neuro-oncologic studies.

Previously, the RANO-PRO working group performed a systematic review to identify all PRO measures used in brain tumor studies. A total of 215 unique PRO measures were identified, of which the majority (70%) were used only once or twice.^16^ The unique identified PRO measures not only included instruments specifically designed for brain tumor patients but also cancer-specific and generic instruments, as well as instruments developed for indications other than cancer (eg, epilepsy or depression). Whether these measures exhibit good content validity or coverage (ie, the degree to which the content of an instrument is an adequate reflection of the construct to be measured^14^) for the primary and secondary brain tumor populations remains to be investigated. This study aimed to establish the content validity (coverage) of these identified PRO measures (*n* = 215) used in primary and secondary brain tumor patient populations.

## Methods

### Framework

The framework of the WHO International Classification of Functioning, Disability, and Health (ICF) was used to establish which aspects of functioning are relevant for brain tumor patients.^17^ The WHO ICF framework refers to the patient’s functioning at 3 distinct levels. The most basic level is a patient’s impairment in body function, for example, a visual impairment. The second level of functioning refers to the consequences of the patient’s impairment in their daily activities. This means that the patient with a visual impairment is no longer able to drive a car. The third and highest level of functioning, so-called participation restrictions, reflects the way the dysfunction affects the patient’s well-being and social interaction. In line with the example, the patient who cannot drive a car may not be able to go to work or attend social activities.

Although the framework covers a wide range of aspects of functioning, from body functions to activities and participation, and while it has been used in other diseases, this is its first application in the primary and secondary brain tumor populations.

### Survey

Based on the WHO ICF framework, a survey was developed for patients, proxies, and healthcare professionals (HCPs) to rate the relevance of the impairments, activity limitations, and participation restrictions described in the WHO ICF framework. We formulated specific decision rules to determine the inclusion of WHO ICF categories in the survey (see Supplementary File 1). For example, only functions that can be assessed with a PRO measure (eg, appetite) were eligible, as opposed to hematological system functions such as blood cell count.

Each item in the survey (*n* = 148, complete survey provided in Supplementary File 2) was scored on a 4-point Likert scale, ranging from not at all (score = 0) to very much (score = 3), reflecting how relevant each issue was for the patient in the past year. Relevance may comprise the frequency with which an issue occurred, but also the severity of the issue. If an item was not applicable or not an issue to the patient’s situation, respondents were instructed to answer “not at all relevant” (score = 0). In addition, 1 open question was added in which respondents could indicate whether there were additional relevant symptoms or issues related to their daily activities or quality of life that were not mentioned in the survey. Several methods of assessment administration were used, depending on the target population: (1) completion of the survey on paper (patients and proxies recruited via outpatient clinics), (2) completion of the survey via the electronic data capture system Castor (patients and proxies recruited via outpatient clinics), or (3) completion of the survey through the electronic system SurveyMonkey (patients, proxies, and HCPs approached via international/national networks). Every item on the survey was assessed as an issue that happened during the past year. This time period was chosen to capture issues that are caused by the tumor as well as its treatment, both acute and long-term, while limiting the impact of recall bias.

### Study Population

Adults with different brain tumor types, their proxies, and HCPs involved in the care of brain tumor patients were invited to participate. Brain tumor patients considered eligible were adults (≥18 years) diagnosed with glioma, primary central nervous system lymphoma (PCNSL), meningioma, or intracranial brain metastasis at any stage of their disease trajectory. Patients were instructed to invite a proxy to participate and were asked to choose a person that has sufficient contact with them to be able to reliably complete a survey on the level of functioning and well-being of the patient. Proxies could be a spouse, partner, family member, or close friend of the patient. HCPs considered eligible were neurologists/neuro-oncologists, neurosurgeons, medical oncologists, radiation oncologists, neuropsychologists, and specialist nurses in neuro-oncology who had at least 2 years of experience treating brain tumor patients or treating ≥20 brain tumor patients per year. Patients and proxies were recruited via (1) outpatient clinics of 5 hospitals in The Netherlands (Haaglanden Medical Center and Leiden University Medical Center), the United States (UCSF Medical Center), India (Apollo Proton Cancer Center), and Switzerland (University Hospital Zurich), and (2) through the network of the International Brain Tumor Alliance (IBTA) based in the United Kingdom. HCPs were approached through national and international professional networks, including the European Association of Neuro-Oncology (EANO). Additionally, sociodemographic and clinical variables were collected from participants through medical records (for patients recruited at the outpatient clinics) or through a study-specific questionnaire (for patients, proxies, and HCPs approached via SurveyMonkey). Written informed consent was required before participation. The institutional review board of Leiden University Medical Center gave approval for the study.

### Statistical Analysis

Descriptive statistics were used to describe the selection procedure of items for the survey, the population characteristics, and the responses to the survey items (eg, means with standard deviations and numbers with percentages). The 4-point Likert scale of the survey items was categorized into binary variables of not applicable or not an issue (score 0) versus an issue (score 1-3), and the percentage of participants in each category was calculated. If ≥25% of the patients, ≥25% of the proxies, or ≥50% of the HCPs considered an item as an issue (score 1-3), the survey item was listed as a relevant item. A cutoff of ≥25% was chosen for patients and their proxies because of disease heterogeneity (eg, based on tumor characteristics such as location, patients are likely to rate different items as relevant). A sensitivity analysis was performed with a score of 0-1 as not relevant and a score of 2-3 as relevant.

To gain insight into the agreement between patients and proxies regarding issues that are relevant to patients with brain tumors, agreement was evaluated for patient-proxy dyads who were recruited via the outpatient clinic of the 5 participating hospitals. First, agreement was assessed using the 4-point Likert score (0, 1, 2, and 3), and next, it was categorized (not applicable/not an issue [score 0] versus an issue [score 1-3]). Agreement in responses between patients and proxies was defined as patients and proxies providing the exact same score and was assessed using weighted Cohen’s kappa and Cohen’s kappa for the 4-point Likert score (0, 1, 2, and 3) and the categorized score (score 0 versus score 1-3), respectively. Estimates between 0.41 and 0.60 represent moderate agreement, 0.61 and 0.80 substantial agreement, and between 0.81 and 0.99 excellent agreement.^18^ Statistics were performed using IBM SPSS Statistics for Windows.

Qualitative data were coded manually by 2 independent reviewers, and disagreement was resolved by consensus. If a new issue (ie, not considered in the survey) was reported by at least 5% of the patients, proxies, or HCPs, the item was considered relevant.

### Linking

The standardized ICF linking rules^19^ were used to link the items in the 215 identified PRO measures to the ICF categories and thereafter to items that were considered relevant in the survey. The percentage of items in a PRO measure that could be considered relevant was calculated in 3 ways, as some items in a questionnaire comprised multiple constructs (eg, in the item “Doze off or fall asleep while watching TV,” 2 aspects are mentioned, which are falling asleep and watching TV). First, an item was considered relevant if the item contained at least 1 relevant aspect (analysis A). Next, an item was considered relevant if at least 50% of the aspects in an item were considered relevant (analysis B), and lastly, an item was considered relevant if all aspects in the item were considered relevant according to our definition (analysis C). Next, the level of coverage was determined for each analytical method. Low content coverage was a priori defined as <50.0% of the items in the questionnaire being relevant, moderate coverage was defined as ≥50.0%-79.9% of the items in the questionnaire being relevant, and sufficient content coverage was defined as ≥80.0% of the items in the questionnaire being relevant. Beforehand, the availability of an existing PRO measure for each issue that was considered relevant was assessed.

## Results

### Construction of the Survey

The WHO ICF comprises 1031 categories, including (sub)headings. After excluding the (sub)headings and items for children, a total of 833 categories remained and were further examined. Of these, 320 (38%) categories were considered not specific enough (eg, “mobility of joint functions, other specified”), and 5 (0.6%) categories were considered too difficult to transform into a PRO item (eg, “acquiring basic concepts”). Out of the remaining 508 categories, 63 categories were considered separate items, and 445 categories were combined into 85 single distinct items. Therefore, the final survey consisted of 148 (63 + 85) items (see Supplementary File 2).

### Study Population

In total, 114 patients, 71 proxies, and 65 HCPs completed the survey; their sociodemographic and clinical characteristics were described in Table 1. Most patients and proxies (89.2% and 94.4%, respectively) were recruited via outpatient clinics, while all HCPs participated via SurveyMonkey.

**Table 1.** Sociodemographic and Clinical Variables of Patients, Healthcare Professionals, and Proxies Participating in the Survey

Type of participant	Patients	Proxies	HCPs
Number of participants	114	71	65
Age, mean (SD) years	55.9 (13.9)	55.9 (13.8)	47.0 (1.3)
Sex (*n*, %) Male Female Missing	64 (56.1) 49 (43.0) 1 (0.9)	23 (32.4) 46 (64.8) 2 (2.8)	31 (47.7) 33 (50.8) 1 (1.5)
HCP experience (years), *n* (%) <2 ≥2	NA NA	NA NA	2 (3.1) 63 (96.9)
Type of HCP, *n* (%) Neurologist/neuro-oncologist Neurosurgeon Medical oncologist Radiation oncologist Neuropsychologist Nurse specialist Others^a^	NA NA NA NA NA NA NA	NA NA NA NA NA NA NA	9 (13.8) 22 (33.8) 10 (15.4) 14 (21.5) 3 (4.6) 3 (4.6) 4 (6.2)
Educational level, *n* (%)^b^ Lower [1-4] Higher [5-8] Missing	50 (43.9) 57 (50.0) 7 (6.1)	32 (45.1) 37 (52.1) 2 (2.8)	NA NA NA
Relationship status, *n* (%) Single Partner	19 (16.7) 95 (83.3)	4 (5.6) 67 (94.4)	NA NA
Relationship with patients, *n* (%) Partner Siblings Child Parent Missing	NA NA NA NA NA	54 (76.1) 4 (5.6) 4 (5.6) 5 (7.0) 4 (5.6)	NA NA NA NA NA
Method of data collection, *n* (%) Outpatient clinic SurveyMonkey	107 (89.2) 13 (10.8)	67 (94.4) 4 (5.6)	— 65 (100)
Living arrangements, *n* (%) Living with a partner Living with a partner and children Living alone Other Missing	49 (43.0) 35 (30.7) 14 (12.3) 14 (12.3) 2 (1.8)	35 (49.3) 24 (33.8) 6 (8.5) 5 (7.0) 1 (1.4)	NA NA NA NA NA
Employment status, *n* (%) Full-time Part-time Retired Unemployed due to illness Other Missing	25 (21.9) 16 (14.0) 30 (26.3) 21 (18.4) 19 (16.7) 3 (2.6)	22 (31.0) 21 (29.6) 19 (26.8) — 8 (11.3) 1 (1.4)	NA NA NA NA NA NA
Tumor type, *n* (%) Glioma Glioblastoma Non-glioblastoma Unknown Meningioma Grade I Grade 2 Grade 3 Unknown Brain metastasis Single metastasis Multiple metastases PCNSL Missing	94 (82.5) 40 (42.6) 52 (55.3) 2 (2.1) 12 (10.5) 5 (41.7) 1 (8.3) 2 (16.7) 4 (33.3) 7 (6.1) 1 (14.3) 6 (85.7) 0 (0.0) 1 (0.9)	NA NA NA NA NA NA NA NA NA NA NA NA NA NA	NA NA NA NA NA NA NA NA NA NA NA NA NA NA
Previous or current treatment, *n* (%)^c^ Resection Radiotherapy Chemotherapy Other (eg, immunotherapy, targeted therapy, tumor-treating fields)	95 (83.3) 88 (77.2) 76 (66.7) 22 (19.3)	NA NA NA NA	NA NA NA NA
KPS, *n* (%) ≤80 >80 Missing ≤70 >70	48 (38.6) 51 (41.2) 25 (20.2) 14 (11.2) 85 (68.6)	NA NA NA NA NA	NA NA NA NA NA
Current disease status, *n* (%) Stable Active Missing	81 (71.7) 32 (28.3) 1 (0.9)	NA NA NA	NA NA NA
Current anti-seizure medication, *n* (%) No Yes Missing	49 (43.0) 52 (45.6) 13 (11.4)	NA NA NA	NA NA NA
Current dexamethasone use, *n* (%) No Yes Missing	88 (77.2) 11 (9.6) 15 (13.2)	NA NA NA	NA NA NA

Abbreviations: HCP, healthcare professional; SD, standard deviation; KPS, Karnofsky Performance Status; NA, not applicable; PCNSL, primary central nervous system lymphoma; TTF, tumor treating fields.

^a^Others: clinical oncologist, neuro-oncology radiographer, pediatric neuro-oncologist, research coordinator.

^b^Educational level according to the International Standard Classification of Education: a score of 1-4 was considered a lower level of education and a score of 5-8 a higher level of education.

^c^Multiple options are possible.

In general, most patients were male (56.1%) and had good performance status (41.2% with a KPS > 80). While none of the patients had a diagnosis of PCNSL, the majority of the patients were diagnosed with a glioma (82.5%). Proxies were mostly female (64.8%) and were the spouse or partner of the patient in 94.4%. HCPs were most often neurosurgeons (33.8%) or radiation oncologists (21.5%).

### Relevance of Items

In total, 56 out of the 148 (37.8%) items in the survey were considered relevant for patients with brain tumors according to our definition (see Table 2 for an overview of items that are considered relevant). A total of 117 symptoms or issues were reported by the participants in the open-ended section of the survey. Nevertheless, of these 117 reported issues, 101 (86.3%) were already covered in the survey, while a few issues mentioned were unclear, not definable, and not covered. For instance, 7 (6.0%) items were unclear, not covered, and not considered an issue (eg, sugar flotation and age); 4 (3.7%) were not covered and not definable (eg, personal weakness and indicating boundaries). Although 5 (4.3%) participants mentioned that seizures were not covered by the survey, this did not exceed our threshold of 5%. Therefore, no items in addition to the 56 items identified were considered.

**Table 2.** Items in the Survey That Were Considered Relevant for Patients with Brain Tumors (*n* = 56), Defined as ≥25% of the Patients, ≥25% of the Proxies, or ≥50% of the Healthcare Professionals Rating the Item as Scores 1-3

Survey items	Reliability test for patient—proxy dyads			Percentage of patients, proxies, and healthcare professionals that reported an item in the survey as an issue relevant to patients (scores 1-3)						
				Main analysis: not relevant (score 0) versus relevant (scores 1-3)				Sensitivity analysis: not relevant (scores 0-1) versus relevant (scores 2-3)		
	Estimand^a^	Analysis A#	Analysis B#	Relevant (yes vs no)	% of Patients	% of Proxies	% of HCPs	% of Patients	% of Proxies	% of HCPs
Item 1: Change in consciousness (ie, alertness, awareness)?	Kappa Agreement *n*(%) AC	0.44 39 (65.0) 60	0.60 48 (80.0) 60	No Yes AC	46.0 54.0 113	47.9 52.1 71	3.1 96.9 65	80.4 19.6 113	85.9 14.1 71	23.1 76.9 65
Item 2: Problems with your orientation?	Kappa Agreement *n*(%) AC	0.40 41 (68.3) 60	0.44 44 (73.3) 60	No Yes AC	55.4 44.6 112	54.9 45.1 71	0.00 100.0 64	84.8 15.2 112	88.7 11.3 71	30.3 79.7 64
Item 3: Change in your behavior/personality (eg, inappropriate language or lack of empathy)?	Kappa Agreement *n*(%) AC	0.31 38 (63.3) 60	0.48 46 (76.7) 46	No Yes AC	59.3 40.7 113	54.3 45.7 70	0.00 100.0 64	91.2 8.8 113	81.4 18.6 70	14.1 85.9 64
Item 4: Lack of energy?	Kappa Agreement *n*(%) AC	0.46 30 (50.0) 60	0.50 49 (81.7) 60	No Yes AC	19.4 80.4 112	25.4 47.9 71	0.00 100.0 65	65.2 34.8 112	56.3 43.7 71	16.9 83.1 65
Item 5: Lack of motivation?	Kappa Agreement *n*(%) AC	0.47 36 (60.0) 60	0.70 51 (85.0) 60	No Yes AC	40.2 59.8 112	52.1 47.9 71	1.5 98.5 65	73.2 26.8 112	74.6 25.4 71	33.8 66.2 65
Item 6: Lack of appetite	Kappa Agreement *n*(%) AC	0.48 42 (72.4) 58	0.52 46 (79.3) 58	No Yes AC	67.9 32.1 112	71.0 29.0 69	12.3 98.5 65	84.8 15.2 112	85.8 14.5 69	63.1 36.9 65
Item 8: Problems sleeping (eg, amount or quality of sleep)?	Kappa Agreement *n*(%) AC	0.63 39 (67.2) 58	0.68 49 (84.5) 58	No Yes AC	39.6 60.4 111	37.1 62.9 70	3.1 96.9 65	74.8 25.2 111	80.0 20.0 70	27.7 72.3 65
Item 9: Difficulty concentrating?	Kappa Agreement *n*(%) AC	0.37 26 (44.8) 58	0.41 46 (79.3) 58	No Yes AC	23.4 76.6 111	20.3 72.9 70	1.6 96.9 64	66.7 33.3 111	59.4 40.6 70	12.5 87.5 64
Item 10: Difficulty remembering things?	Kappa Agreement *n*(%) AC	0.41 29 (50.0) 58	0.60 49 (84.5) 58	No Yes AC	21.6 78.4 111	27.1 72.7 70	0.00 100.0 63	64.0 36.0 111	60.0 40.0 70	11.1 88.9 63
Item 13: Problems controlling your emotions (eg, control of anger)?	Kappa Agreement *n*(%) AC	0.50 44 (77.2) 57	0.61 47 (82.5) 57	No Yes AC	55.0 45.0 111	61.8 38.8 68	3.2 96.8 63	88.3 11.7 111	94.1 5.9 68	36.5 63.5 63
Item 14: Change in your emotions (eg, more or less anxiety or anger)?	Kappa Agreement *n*(%) AC	0.36 34 (60.7) 56	0.35 38 (67.9) 56	No Yes AC	45.4 54.6 108	50.0 50.0 68	1.6 98.4 63	84.3 15.7 108	79.4 20.6 68	33.3 66.7 63
Item 15: Problems thinking (eg, thinking slowly or having uncontrollable thoughts)?	Kappa Agreement *n*(%) AC	0.31 29 (50.9) 57	0.37 39 (68.4) 57	No Yes AC	47.7 52.3 109	50.7 49.3 64	0.00 100.0 64	81.7 18.3 109	79.7 20.3 64	23.4 76.8 64
Item 16: Problems with organization and planning (eg, organizing a dinner)?	Kappa Agreement *n*(%) AC	0.44 32 (54.2) 59	0.42 42 (71.2) 59	No Yes AC	48.6 51.4 111	42.6 57.4 68	0.00 100.0 64	79.3 20.7 111	70.6 29.4 68	25.0 75.0 64
Item 17: Problems managing your time?	Kappa Agreement *n*(%) AC	0.51 40 (67.8) 59	0.53 46 (78.0) 59	No Yes AC	57.3 42.3 110	60.3 39.7 68	3.2 96.8 63	84.5 15.5 110	83.8 16.2 68	49.2 50.8 63
Item 18: Problems switching between thoughts (eg, when problem-solving)?	Kappa Agreement *n*(%) AC	0.47 34 (58.6) 58	0.55 45 (77.6) 58	No Yes AC	47.7 52.3 109	44.1 55.9 68	4.7 95.3 64	83.5 16.5 109	82.4 17.6 68	39.1 60.9 64
Item 19: Lack of insight into your behavior?	Kappa Agreement *n*(%) AC	0.24 36 (63.2) 57	0.35 42 (73.7) 57	No Yes AC	71.8 28.2 110	58.2 41.8 67	4.7 95.3 64	95.5 4.5 110	83.6 16.4 67	42.2 57.9 64
Item 21: Difficulty solving problems?	Kappa Agreement *n*(%) AC	0.39 36 (62.1) 58	0.44 42 (72.4) 58	No Yes AC	53.2 46.8 111	58.7 41.8 67	0.00 100.0 64	91.0 9.0 111	89.6 10.4 67	28.1 71.9 64
Item 23: Problems expressing language (ie, spoken or written messages, or sign or body language)?	Kappa Agreement *n*(%) AC	0.48 44 (74.6) 59	0.51 47 (79.7) 59	No Yes AC	66.7 33.3 111	70.1 29.9 67	0.00 100.0 64	91.0 9.0 111	92.5 7.5 67	21.9 78.1 64
Item 29: Any other problems with the eyes (eg, tired, dry, or itchy eyes)?	Kappa Agreement *n*(%) AC	0.56 42 (70.0) 60	0.48 45 (75.0) 60	No Yes AC	58.4 41.6 113	66.2 33.8 71	20.0 80.0 60	90.3 9.7 113	91.5 8.5 71	71.7 28.3 60
Item 30: Problems with hearing?	Kappa Agreement *n*(%) AC	0.35 25 (48.1) 52	0.42 37 (71.2) 52	No Yes AC	60.9 39.1 110	52.3 47.7 65	18.3 81.9 60	89.1 10.9 110	80.0 20.0 65	63.3 36.7 60
Item 32: Problems maintaining balance while standing or moving?	Kappa Agreement *n*(%) AC	0.55 39 (66.1) 59	0.59 47 (79.7) 59	No Yes AC	50.9 49.1 110	72.5 27.5 69	1.7 98.3 60	80.0 20.0 110	85.7 14.3 69	30.0 70.0 60
Item 33: Dizziness?	Kappa Agreement *n*(%) AC	0.30 39 (68.4) 57	0.29 41 (71.9) 57	No Yes AC	62.7 37.3 110	72.5 27.5 70	1.7 98.3 60	90.0 10.0 110	97.1 2.9 70	36.7 63.3 60
Item 37: Change in sensitivity to temperature?	Kappa Agreement *n*(%) AC	0.54 44 (77.2) 57	0.68 50 (87.7) 57	No Yes AC	71.2 26.1 111	67.8 32.4 67	38.3 61.7 60	88.3 11.7 111	85.3 14.7 67	78.3 21.7 60
Item 41: Pain in one body part (eg, headache or back pain)?	Kappa Agreement *n*(%) AC	0.45 38 (67.9) 56	0.64 46 (82.1) 56	No Yes AC	47.3 52.7 110	60.3 39.7 68	5.0 95.5 60	84.5 15.5 110	82.4 17.6 68	25.0 75.0 60
Item 42: Pain in multiple body parts (eg, headache and back pain)?	Kappa Agreement *n*(%) AC	0.45 39 (72.2) 54	0.54 43 (79.6) 54	No Yes AC	66.1 33.9 109	67.2 32.8 67	11.7 88.3 60	88.1 11.9 109	86.6 13.4 67	55.0 45.0 60
Item 43: Radiating pain in a specific region?	Kappa Agreement *n*(%) AC	0.38 39 (72.2) 54	0.50 43 (79.6) 54	No Yes AC	67.0 33.0 109	71.6 28.4 67	18.3 81.7 60	95.4 4.6 109	94.0 6.0 67	51.7 48.3 60
Item 45: Problems with speech (eg, tempo, slurring words, stuttering, or articulating)?	Kappa Agreement *n*(%) AC	0.25 39 (65.0) 60	0.23^a^ 42 (70.0) 60	No Yes AC	65.2 34.8 112	69.0 31.0 71	0.0 100.0 59	89.3 10.7 112	91.5 8.5 71	22.0 78.0 59
Item 54: Reduced tolerance to physical exercise (eg, feeling out of breath or more easily fatigued)?	Kappa Agreement *n*(%) AC	0.48 33 (61.1) 54	0.40 38 (70.4) 54	No Yes AC	46.8 53.2 111	53.1 56.9 64	5.1 94.9 59	73.9 26.1 111	82.8 17.2 64	33.9 66.1 59
Item 61: Problems with bowel movements (eg, change in consistency or frequency of bowel movements)?	Kappa Agreement *n*(%) AC	0.36 32 (57.1) 56	0.17^b^ 34 (60.7) 56	No Yes AC	56.9 43.1 109	66.7 33.3 66	12.5 87.5 56	85.3 14.7 109	86.4 13.6 66	48.2 51.8 56
Item 62: Problems maintaining your body weight?	Kappa Agreement *n*(%) AC	0.47 39 (69.6) 56	0.55 44 (78.6) 56	No Yes AC	56.4 43.6 110	62.7 37.3 67	19.6 80.4 56	87.2 12.7 110	85.1 14.9 67	58.9 41.1 56
Item 67: Constantly feeling too hot or too cold?	Kappa Agreement *n*(%) AC	0.36 34 (63.0) 54	0.38 40 (74.1) 54	No Yes AC	68.2 31.8 110	71.9 28.1 64	49.1 50.9 55	89.1 10.9 110	87.5 12.5 64	83.6 16.4 55
Item 68: Problems with urination (eg, changes in frequency or incontinence)?	Kappa Agreement *n*(%) AC	0.33 36 (60.0) 60	0.40 44 (73.3) 60	No Yes AC	62.5 37.8 112	68.9 31.4 70	7.3 92.7 55	87.5 12.5 112	87.1 12.9 70	56.4 43.6 55
Item 70: Problems with sexual activity (eg, change in sexual interest or trouble reaching an orgasm)?	Kappa Agreement *n*(%) AC	0.35 30 (50.8) 59	0.39 41 (69.5) 59	No Yes AC	57.3 42.7 110	49.3 50.7 69	10.9 89.1 55	78.2 21.8 110	66.7 33.3 69	60.0 40.0 55
Item 74: Problems with your joints (eg, ease of movement or dislocation)?	Kappa Agreement *n*(%) AC	0.46 43 (71.7) 60	0.49 48 (80.0) 60	No Yes AC	67.0 33.0 112	72.9 27.1 70	25.5 74.5 55	86.6 13.4 112	87.1 12.9 70	67.3 32.7 55
Item 76: Tension in muscles?	Kappa Agreement *n*(%) AC	0.42 40 (67.8) 59	0.47 45 (76.3) 59	No Yes AC	64.5 35.5 110	66.2 33.8 68	20.0 80.0 55	86.4 13.6 110	92.6 7.4 68	72.7 27.3 55
Item 79: Distorted walking pattern (eg, swaying or stiff walking pattern)?	Kappa Agreement *n*(%) AC	0.53 47 (81.0) 58	0.74 52 (89.7) 58	No Yes AC	66.1 33.9 109	73.5 26.5 68	1.8 98.2 55	88.1 11.9 109	91.2 8.8 68	30.9 69.1 55
Item 80: Sensation of muscle stiffness?	Kappa Agreement *n*(%) AC	0.56 45 (76.3) 59	0.69 52 (88.1) 59	No Yes AC	70.9 29.1 110	72.1 27.9 68	27.3 72.7 55	90.9 10.0 110	92.6 7.4 68	63.6 36.4 55
Item 81: Problems with your skin (eg, change in pigmentation, sensitivity to sunlight, or more sweating than usual)?	Kappa Agreement *n*(%) AC	0.37 44 (72.1) 61	0.43 48 (78.7) 61	No Yes AC	75.0 25.0 112	71.8 28.2 71	34.5 65.5 55	95.5 4.5 112	95.8 4.2 71	85.5 14.5 55
Item 82: Unpleasant sensations of the skin (eg, itching, burning, or tingling)?	Kappa Agreement *n*(%) AC	0.29 40 (67.8) 59	0.32 43 (72.9) 59	No Yes AC	67.3 32.7 110	71.4 28.6 70	27.3 72.7 55	88.2 11.8 110	97.1 2.9 70	76.4 23.6 55
Item 83: Problems with your hair (eg, change in growth, color, or location of hair)?	Kappa Agreement *n*(%) AC	0.39 44 (74.6) 59	0.56 48 (81.4) 59	No Yes AC	64.9 35.1 111	67.6 32.4 71	25.5 74.5 55	95.5 4.5 111	90.1 9.9 71	65.5 34.5 55
Item 91: Problems obtaining new information (eg, asking for facts or names of people)?	Kappa Agreement *n*(%) AC	0.41 42 (73.7) 57	0.47 43 (75.4) 57	No Yes AC	55.1 44.9 107	68.7 31.3 67	1.8 98.2 55	88.8 11.2 107	95.5 4.5 67	30.9 69.1 55
Item 97: Problems learning new skills (eg, playing a new game)?	Kappa Agreement *n*(%) AC	0.35 38 (10.9) 55	0.39 42 (76.4) 55	No Yes AC	67.0 33.0 106	70.3 29.7 64	5.5 94.5 55	87.7 12.3 106	93.8 6.3 64	56.4 43.6 55
Item 98: Problems making decisions?	Kappa Agreement *n*(%) AC	0.49 40 (70.2) 57	0.60 46 (80.7) 57	No Yes AC	50.9 49.1 108	59.1 40.9 66	0.00 100.0 55	79.6 20.4 108	83.3 16.7 66	23.6 76.4 55
Item 100: Problems doing several different things at the same time?	Kappa Agreement *n*(%) AC	0.29 28 (47.5) 59	0.35 40 (67.8) 59	No Yes AC	37.2 62.8 113	39.7 60.3 68	0.00 100.0 55	90.3 9.7 113	75.0 25.0 68	21.8 78.2 55
Item 101: Problems functioning independently in daily life?	Kappa Agreement *n*(%) AC	0.47 43 (71.7) 60	0.51 48 (80.0) 60	No Yes AC	66.4 33.6 113	67.6 32.4 68	0.00 100.0 54	88.1 11.9 113	91.2 8.8 68	13.0 87.0 54
Item 103: Problems handling stress?	Kappa Agreement *n*(%) AC	0.42 32 (54.2) 59	0.45 43 (72.9) 59	No Yes AC	38.2 61.8 110	39.1 60.9 69	1.8 98.2 55	71.8 28.2 110	79.7 20.3 69	27.3 72.7 55
Item 104: Problems having a conversation (eg, starting, continuing, or ending a conversation with one person or many people)?	Kappa Agreement *n*(%) AC	0.19^b^ 43 (70.5) 61	0.29 45 (73.8) 61	No Yes AC	99.7 33.3 114	71.4 28.6 70	0.00 100.0 54	85.1 14.9 114	92.9 7.1 70	38.9 61.1 54
Item 105: Problems having a discussion (eg, starting, continuing, or ending a discussion with one person or many people)?	Kappa Agreement *n*(%) AC	0.29 37 (61.7) 60	0.38 43 (71.1) 60	No Yes AC	60.2 39.8 113	65.7 34.3 70	1.9 98.1 54	81.4 18.6 113	90.0 10.0 70	33.3 66.7 54
Item 106: Problems using communication devices (eg, telephone or computer)?	Kappa Agreement *n*(%) AC	0.35 43 (71.1) 60	0.33 44 (73.3) 60	No Yes AC	69.9 30.1 113	73.5 26.5 68	3.7 96.3 54	92.9 7.1 113	97.0 2.9 68	42.6 57.4 54
Item 107: Difficulty changing your body position (eg, lying down, sitting down, or standing up)?	Kappa Agreement *n*(%) AC	0.57 44 (73.3) 60	0.51 48 (80.0) 60	No Yes AC	62.3 37.7 114	72.1 27.9 68	3.8 96.2 52	88.6 11.4 114	92.6 7.4 68	48.1 51.9 52
Item 116: Difficulty moving around by means other than walking (eg, climbing stairs, swimming, or running)?	Kappa Agreement *n*(%) AC	0.52 42 (79.2) 53	0.72 47 53	No Yes AC	63.6 36.4 107	70.3 29.7 64	3.9 96.1 51	82.2 17.8 53	89.1 10.9 64	31.4 68.6 51
Item 119: Problems driving a vehicle (eg, a car or bicycle)?	Kappa Agreement *n*(%) AC	0.49 38 (73.1) 52	0.48 40 (76.9) 52	No Yes AC	67.0 33.0 106	58.7 41.3 63	5.7 94.3 53	77.4 22.6 106	74.6 25.4 63	26.4 73.6 53
Item 142: Problems engaging in paid work?	Kappa Agreement *n*(%) AC	0.57 39 (76.5) 51	0.64 43 (84.3) 51	No Yes AC	63.2 36.8 106	66.7 33.3 63	7.7 92.3 52	79.2 20.3 106	77.8 22.2 63	19.2 80.8 52
Item 144: Problems doing your personal finances?	Kappa Agreement *n*(%) AC	0.45 43 (74.1) 58	0.55 48 (82.8) 58	No Yes AC	73.0 27.0 111	68.2 31.8 66	7.8 92.2 51	91.0 9.0 111	84.8 15.2 66	33.3 66.7 51
Item 145: Problems participating in community activities (eg, clubs or organizations)?	Kappa Agreement *n*(%) AC	0.27 42 (71.2) 59	0.35 45 (76.3) 59	No Yes AC	69.1 30.9 110	73.1 26.9 67	11.8 88.2 51	86.4 13.6 110	86.6 13.4 67	49.0 51.0 51
Item 146: Problems participating in recreation and leisure activities (eg, sports or hobbies)?	Kappa Agreement *n*(%) AC	0.46 43 (74.1) 58	0.52 47 (81.0) 58	No Yes AC	68.5 31.5 111	66.7 33.3 69	12.8 87.2 51	85.6 14.4 111	84.1 15.9 69	51.1 48.9 51

Abbreviations: HCP, healthcare professionals; AC, available case. #Analysis A: Agreement assessed using the 4-point Likert response score ranging from 0 to 3, using weighted kappa. #Analysis B: Agreement assessed using no issue not relevant/not applicable (score 0) versus relevant issue (scores 1-3) as outcomes by means of standard kappa.

^a^Agreement *n*(%) is the observed difference between patients and proxies, with kappa and weighted kappa estimated for analyses A and B, respectively.

^b^Kappa or weighted kappa was not significant at a *P*-value >.05.

**Figure 1.**
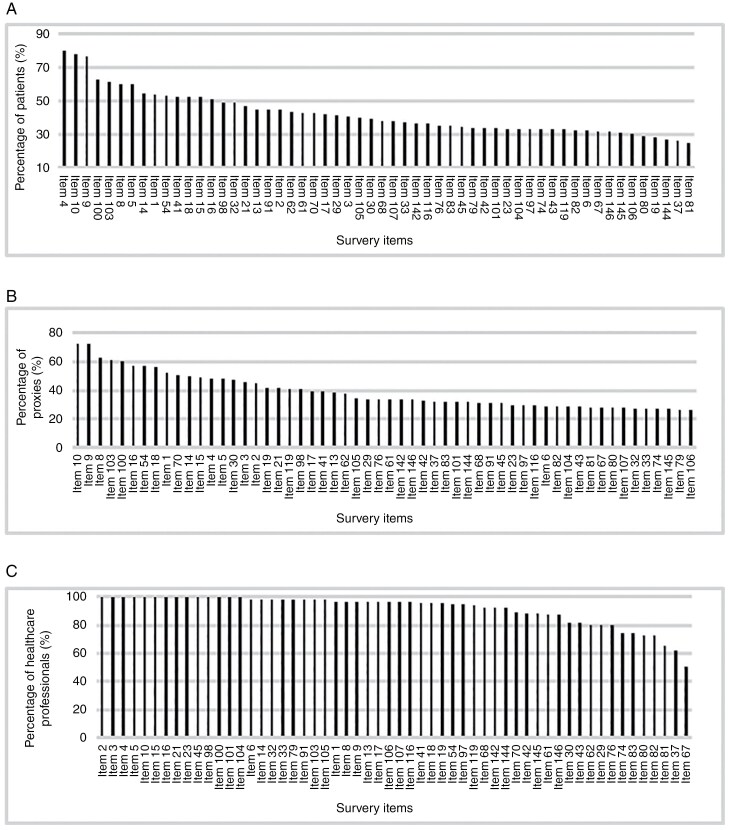
Percentage of patients (A), proxies (B), and healthcare professionals (C) that considered 56 items in the survey relevant for patients with brain tumors.

The 5 issues considered most relevant by patients were lack of energy (item #4, 80.4%), difficulty concentrating (item #9, 76.6%), difficulty remembering (item #10, 78.4%), problems doing several things at the same time (item #100, 62.8%), and problems handling stress (item #103, 61.8%). Proxies also considered difficulty concentrating (item #9, 72.9%), difficulty remembering (item #10, 72.7%), problems doing several things at the same time (item #100, 60.9%), and problems handling stress (item #103, 60.9%) as relevant, but problems sleeping (item #8, 62.9%) was also in the top 5 items. In total, 16 items were rated relevant by all HCPs. These comprised problems with orientation (#2), change in behavior/personality (#3), lack of energy (#4), difficulty remembering things (#10), problems thinking (#15), problems with organization and planning (#16), difficulty solving problems (#21), problems expressing language (#23), problems in part of the visual field (#28), problems with speech (#45), problems making decisions (#98), problems carrying out a single task independently (#99), problems doing several things at the same time (#100), problems functioning independently in daily life (#101), problems handling responsibilities, eg, taking care of children or going to work (#102) and problems having a conversation (#104) (Figure 1 and Supplementary Table 3).

### Agreement between Patients and Proxies

**Figure 2.**
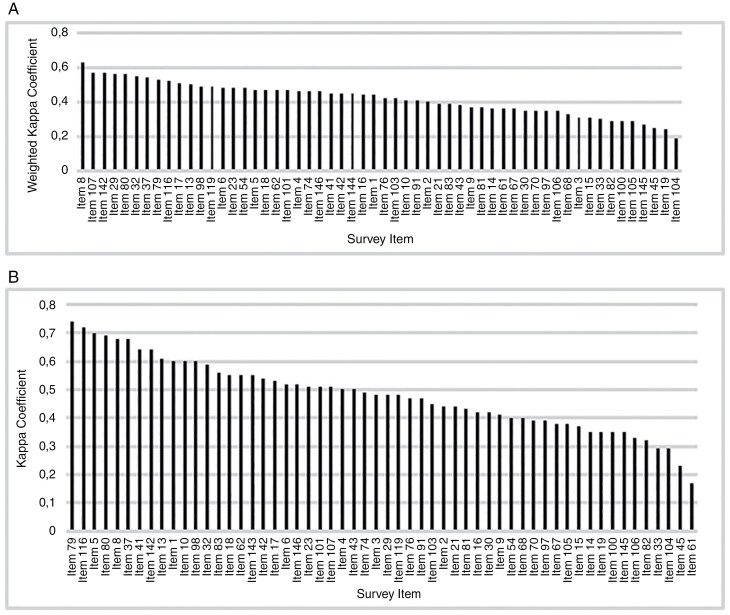
Agreement between 67 patient-proxy dyads for the 56 items in the survey that were considered relevant for patients with brain tumors. (A) Analysis A, where agreement was assessed using the 4-point Likert response score ranging from 0 to 3, with an estimated weighted kappa. (B) Analysis B, where agreement was assessed using a dichotomized scoring (no issue/not relevant/not applicable [score 0] versus issue/relevant/applicable [scores 1-3]) with an estimated kappa. See Supplementary File 2 for the content of the items.

The agreement between patients and proxies in relevance of issues was calculated for 67 patient-proxy dyads. Patients and proxies had moderate to excellent agreement on 72/148 (48.6%) of the items in the survey (weighted kappa ≥ 41). When categorizing the data, patients and proxies had moderate to excellent agreement on 86/148 (58.1%) of the items (kappa ≥ 41) (Supplementary File 3). When considering the 56/148 items that were deemed relevant in this study according to our definition, items #8 (problems sleeping (eg, amount or quality of sleep)?) and #79 (distorted walking patterns) had the highest agreement in analyses A and B, respectively (Figure 2 and Table 2).

### Content Coverage of the Identified PRO Measures

**Figure 3.**
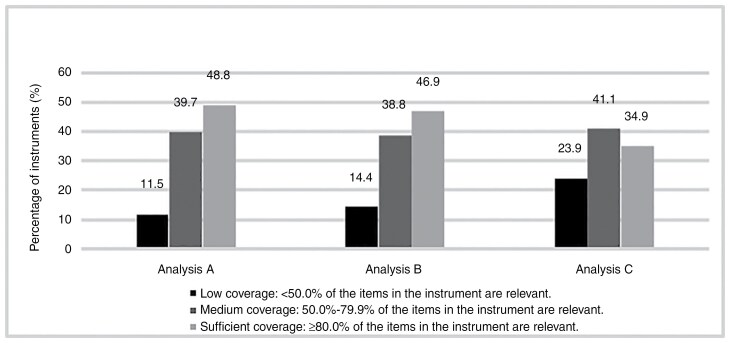
The percentage of PRO measures with low (≤50.0% of the items relevant), medium (50.0%-79.9% of the items relevant), and sufficient (≥80.0% of the items relevant) content coverage. Three ways of calculating the percentage of items in a PRO measure that could be considered relevant were used, as some items in a questionnaire comprised multiple constructs (eg, in the item “Doze off or fall asleep while watching TV,” 2 aspects are mentioned, which are falling asleep and watching TV). First, an item was considered relevant if the item contained at least 1 relevant aspect (analysis A). Next, an item was considered relevant if at least 50% of the aspects in an item were considered relevant (analysis B). Lastly, an item was considered relevant if all aspects of the item were considered relevant according to our definition (analysis C).

Six (2.8%) out of the 215 previously identified PRO measures^16^ could not be retrieved for item linking (BRIEF-A, EBIQ, GSDS, Neuro-QOL, PAIS-SR, and a study-specific questionnaire). Thus, item linking was performed in the remaining 209 PRO measures (Figure 3 and Supplementary Table 4). When at least 1 aspect of an item should be considered relevant (analysis A), 48.8% (102/209) of the identified PRO measures had sufficient content coverage, defined as ≥80% of the items in the questionnaire being relevant. When at least 50% of the aspects in an item should be considered relevant (analysis B), the percentage of PRO measures with sufficient content coverage dropped slightly to 46.9% (98/209). Last, when all aspects of an item should be considered relevant, only 34.9% (73/209) of the PRO measures had sufficient content coverage. Of note, issues that were considered relevant were covered by the existing PRO measures used in primary and secondary brain tumor populations.

## Discussion

The content validity of individual items within PRO measures^16^ identified in the systematic review used in primary and secondary brain tumor patient populations was studied by linking them to the relevant aspects from the WHO ICF framework. In total, 56 aspects of the WHO ICF framework were considered relevant by patients, proxies, or HCPs. The most important aspects mentioned by both patients and proxies were difficulty concentrating, difficulty remembering, problems doing several things at the same time, and problems handling stress. Patients also considered lack of energy as important, while proxies mentioned problems sleeping. HCPs added problems with cognition, such as thinking, organization, and planning, as important aspects, as well as problems with speech, vision, and independent functioning in daily life. Generally, HCPs considered different items as relevant compared to the patients and proxies, with a particular survey item being more often considered a relevant issue by HCPs than by patients and their proxies. This is possibly a result of patients and proxies having reflected on a more personal level, whereas HCPs have reflected on a population level with different patients in mind. Additionally, some items in the questionnaire are more observable to HCPs and proxies than more subjective and personal items such as pain, energy levels, nausea, and emotions. However, if the subjective items are still observable to HCPs, reporting their accurate severity might be difficult for HCPs and proxies, thus leading to differences in the items considered relevant between patients, proxies, and HCPs. When linking these relevant issues to items within the unique 209 available PRO measures, we found that between 34.9% and 48.8% of these PRO measures could be considered as having sufficient content validity depending on our various definitions.

Of the 10 most used measures identified in the systematic review,^16^ only 2 measures exceeded the threshold of ≥80% coverage, namely the Hospital Anxiety and Depression Scale (HADS) and the Beck Depression Inventory-II (BDI-II) questionnaire, each with a content coverage of 100%. The content coverage of the EORTC QLQ-C30, QLQ-BN20, EQ-5D, MDASI-BT, PRO-CTCAE, SF-36, and Fact-Br questionnaires ranged between 50.0% and 66.7%. These findings can be explained by the number of domains assessed in a questionnaire. In contrast to the HADS and BDI-II, which cover only one domain, the other questionnaires are multidimensional, addressing multiple domains. This may explain why the content coverage of these multidimensional questionnaires did not exceed the threshold for sufficient content validity. If certain domains are not considered relevant, then the content validity of that questionnaire is thereby diminished. Moreover, many questionnaires were developed and validated in the cancer patient population or in the general population and are not validated in the primary and secondary brain tumor populations. As a consequence, these questionnaires may address aspects that are not considered relevant for brain tumor populations and thus result in a lower content coverage. Furthermore, long-existing questionnaires may not cover aspects of the side effects and toxicities of more recently developed treatments. Our results emphasize that the selection of the most appropriate instrument should depend on the content that is relevant for the specific setting or research objective. For example, the research objective could be to assess 1 domain, such as emotional wellness, or the multidimensional construct of health-related quality of life, each requiring different instruments. Of note, when selecting an instrument, it is also important to consider other psychometric properties such as reliability and responsiveness, as well as the content validity and practical limitations such as available translations.

Importantly, our results are highly dependent on the methodology we employed. First, the definitions for considering an item as relevant as well as the definition of sufficient content coverage were arbitrary. Although using other definitions may change the results of this study, we believe that our results are valuable as they provide insight into the number of domains in each instrument that are relevant for brain tumor patient populations and guide the choice of an instrument. Second, the study’s sample of patients, proxies, and HCPs was limited in the setting of reduced recruitment during the COVID-19 pandemic. Among HCP respondents, the lack of neuro-oncology nurses and physicians is a potential source of bias. Third, generalizability of the results to the entire brain tumor population might be limited. Although the sampling of patients from various countries and continents provided at least a partially representative sample of the global brain tumor population, the sample size remained relatively small, and patients with glioma were overrepresented, possibly limiting the validity of results for other tumor types. Patients with good health status also seem to be overrepresented, as the percentage of patients with a KPS ≤70 is only 11.2%, and the majority of patients were not on steroids (77.2%). This is common in research, in which patients with a better health status are often overrepresented, as they are probably more willing and able to take part in a survey as compared to patients in worse health status. Moreover, the limited sample size, particularly for certain tumor types, prevented a reliable analysis of issues that are important for a certain group of patients. Thus, subgroup analysis could be an important step to consider in further research. Fourth, although the WHO ICF framework covers a wide range of aspects of functioning, from body functions to activities and participation, and while it is used in other diseases,^20–22^ several aspects that are important to adults with brain tumors are missing. For example, seizure is not covered, although it is a common symptom in this population, as reported in Table 1 and in other studies in patients with brain tumors.^23,24^ Lastly, the linking process was challenging. Some aspects of the items in the PRO measures were not sufficiently specified (eg, “I am frustrated by being too tired to do the things I want to do”), so “do the things I want to do” had to be recorded as “not definable.” In other cases, the meaning of a certain aspect of an item could be debated as to what was exactly meant and subsequently what would be the best category to link the item to (eg, “Other people being aware of your private thoughts”). Finally, in an open-ended section of the survey, a few issues mentioned by the patients were unclear and not definable (eg, sugar flotation, personal weakness, and indicating boundaries), and it could be argued what the patients meant exactly, limiting their classification into levels of relevancy. Future projects could explore the use of machine learning methods that fully and automatically link information from PRO measures to aspects of the WHO ICF framework.^25^

Despite these limitations, the results of this study suggest that the use of static questionnaires with a fixed number of items may not meet the current needs of brain tumor patients, HCPs, and other stakeholders, such as regulators. With a static questionnaire, it may be that several aspects are assessed that are not considered relevant. A more flexible approach in which a standard set of relevant items is complemented with items that are relevant for a certain situation (eg, when assessing a new treatment with a certain toxicity profile) could be a solution. To this end, item libraries, which are large databases comprising single- and multi-item scales, could be used.^26–28^ The Fast Track COA Group defined a core set of symptoms and functions that should be assessed in all tumor-directed treatment trials for high-grade glioma patients.^15^ The functions include physical, role, and social functioning, and the symptoms include pain, perceived cognition, difficulty communicating, seizures, and symptomatic adverse events. These constructs were all considered relevant in our survey, except for seizures, as this was not included in the survey because it was not covered in the WHO ICF. This is a serious limitation given that seizures are commonly occurring in both primary and metastatic brain tumors.

In conclusion, the content validity based on the WHO ICF framework of 209 unique PRO measures was sufficient in less than half of the measures. In particular, the content validity was insufficient in multidimensional measures. Future research should investigate whether a flexible approach in PRO assessment better meets the needs of all stakeholders.

## Supplementary Material

noaf108_suppl_Supplementary_Materials

## Data Availability

Data will be made available upon reasonable request.
