# Supplementary material for: Establishing the content validity of patient-reported outcome measures used in neuro-oncology based on the WHO ICF framework: Part of the RANO-PRO initiative
Source: Neuro Oncol. 2025 Apr 28;27(9):2399–414. doi: 10.1093/neuonc/noaf108 (PMC12526050; doi:10.1093/neuonc/noaf108)
Supplement: noaf108_suppl_Supplementary_Materials [file noaf108_suppl_supplementary_materials.docx]

**Supplemental File 1. Decision rules for the development of the survey**

Link to the WHO ICF: <http://apps.who.int/classifications/icfbrowser/>

Decision rules:

1. No question, name of domain, subcategories specified;

2. No question, item specific for children;

3. No question, item not specific enough;

4. No question, item not measurable with PRO, symptoms covered by other items;

5. No question, item too complicated to measure with PRO;

6. No question, item covered elsewhere;

7. Merged into one question;

8. Merged into one question, items not comprehensive;

9. Separate question, not possible to merge items into one question;

10. Separate question, item not measurable with PRO, symptom(s) not covered by other items.

**Explanation of the decision rules**

To calculate the total number of items of the WHO ICF, only the items at the final-level categories were included and not the domains (which are further subdivided into subdomains and/or categories).

In the following cases items were not counted in the total number of unique items, and therefore not included in the survey:

- Items that were further subdivided into domains and/or categories (decision rule 1);
- Items that were specifically for children (decision rule 2).

In the following cases items were counted in the total number of items, but not included in the survey:

- Items that were not specific enough, such as items that include ‘other specified’ or ‘unspecified’ (decision rule 3);
- Items that were not measurable with a PRO measure, such as ‘production of blood’, and for which the associated symptoms are covered by other questions (decision rule 4);
- Items that were too complicated for a PRO measure, such as ‘abstraction’ (decision rule 5);
- Items that were already covered by other questions in the survey (decision rule 6).

In the following cases items were counted in the total number of items, but merged into one question in the survey:

- Items of a domain/category for which one question was enough to cover all of the items (decision rule 7);
- Items of a domain/category which were not comprehensive for the total domain/category (decision rule 8).

In the following cases items were counted in the total number of items, and included as a separate question in the survey:

- Items of a domain/category for which it was not possible to merge them into one question that covers all the items (decision rule 9);
- Items that were not measurable with a PRO measure, but involving symptoms that were not covered by other questions in the survey. In this case one or multiple symptoms of that item were included in the questionnaire (decision rule 10).

**Supplemental File 2. Survey (patient version)**

Instructions

Below is a list of symptoms and issues related to daily functioning and quality of life that patients with brain tumors sometimes report. Please rate the relevance of the symptoms and issues to your own situation during the past year. Please answer all of the questions yourself by circling the number that best applies to you. There are no ‘’right’’ or ‘’wrong’’ answers. The information that you provide will remain strictly confidential.

Please rate the relevance of the issues addressed in the questionnaire on a 4-point scale:

0 = not at all relevant

1 = a little bit relevant

2 = quite a bit relevant

3 = very relevant

Please note that a symptom or issue should only be rated relevant if it applies to your own situation. You can think of how often a symptom or issue has occurred during the past year, but also how important this symptom or issue is.

Most likely there will be questions that do not apply to your situation. The reason we have included all these questions is that we want to find out if there is anything that we do not yet know about and that we do not normally ask about.

If a question does not apply to your situation, you can answer the question with ‘0 = not at all relevant’.

Some of the questions include examples. These are just examples and the question can still be relevant to you if one or even all of the examples do not apply to your situation.

This is quite an extensive questionnaire. Please take your time to fill in all of the questions. You do not have to answer all the questions at once and can complete the questionnaire later.

How relevant are the following issues to your own situation? Please circle one number per line as it best applies to your situation in the past year.

| **Functions of the brain**   1. Change in consciousness (i.e. alertness, awareness)? 2. Problems with your orientation? 3. Change in your behavior/personality (e.g. inappropriate language or lack of empathy)? 4. Lack of energy? 5. Lack of motivation? 6. Lack of appetite? 7. Craving (i.e. a very strong urge to consume e.g. certain food or drinks)? 8. Problems sleeping (e.g. amount or quality of sleep)? 9. Difficulty concentrating? 10. Difficulty remembering things? 11. Problems carrying out intentional movements (e.g. reaching for and lifting a cup of coffee)? 12. Problems showing appropriate emotions (e.g. no happiness at receiving good news)? 13. Problems controlling your emotions (e.g. control of anger)? 14. Change in your emotions (e.g. more or less anxiety or anger)? 15. Problems thinking (e.g. thinking slowly or having uncontrollable thoughts)? 16. Problems with organization and planning (e.g. organizing a dinner)? 17. Problems managing your time? 18. Problems switching between thoughts (e.g. when problem-solving)? 19. Lack of insight into your behavior? 20. Lack of judgement (e.g. whether it is safe to cross the road)? 21. Difficulty solving problems? 22. Problems understanding language (i.e. spoken or written messages, or sign or body language)? 23. Problems expressing language (i.e. spoken or written messages, or sign or body language)? 24. Difficulty doing calculations? 25. Lack of self-awareness (e.g. body image or identity)? 26. Lack of insight in the length or passage of time?   **Functions of the senses and the sensation of pain**   1. Problems with your vision (e.g. distinguishing forms, color or contrast, or double vision)? 2. Problems in part of your visual field (e.g. partial loss of vision)? 3. Any other problems with the eyes (e.g. tired, dry or itchy eyes)? 4. Problems with hearing? 5. Any other problems with the ears (e.g. irritation, ringing or pressure)? 6. Problems maintaining balance while standing or moving? 7. Dizziness? 8. Change in taste? 9. Change in smell? 10. Change in feeling of touch (e.g. numbness or tingling)? 11. Change in sensitivity to temperature? 12. Change in sensitivity to vibration? 13. Change in sensitivity to pressure? 14. Pain throughout the body? 15. Pain in one body part (e.g. headache **or** back pain)? 16. Pain in multiple body parts (e.g. headache **and** back pain)? 17. Radiating pain in a specific region?   **Functions of producing sounds and speech**   1. Problems with your voice (e.g. loudness or hoarseness)? 2. Problems with speech (e.g. tempo, slurring words, stuttering or articulating)? 3. Problems producing sounds (e.g. singing or humming)?   **Functions of the heart, respiration and exercise tolerance**   1. Problems with your heart rate (e.g. too fast, too slow or irregular)? 2. Leg thrombosis (i.e. red, swollen, painful leg)? 3. Problems with your breathing (e.g. shortness of breath or rapid and deep breathing)? 4. Problems coughing? 5. Problems sneezing? 6. Problems yawning? 7. Dry cough? 8. Reduced tolerance to physical exercise (e.g. feeling out of breath or more easily fatigued)?   **Functions of digestion and metabolism**   1. Problems sucking? 2. Problems biting? 3. Problems chewing? 4. Change in amount of saliva (e.g. drooling or dry mouth)? 5. Problems swallowing? 6. Vomiting? 7. Problems with bowel movements (e.g. change in consistency or frequency of bowel movements)? 8. Problems maintaining your body weight? 9. Nausea? 10. Bloated feeling in your belly? 11. Stomach cramps? 12. Swelling in any part of your body (e.g. ankles, legs, arms, hands or fingers)? 13. Constantly feeling too hot or too cold?   **Functions of urination, sexual activity and procreation**   1. Problems with urination (e.g. changes in frequency or incontinence)? 2. Unpleasant sensations with urination (e.g. incomplete voiding or feeling of fulness of bladder)? 3. Problems with sexual activity (e.g. change in sexual interest or trouble reaching an orgasm)? 4. ***Question for female patients only:*** Problems with your menstruation (e.g. regularity or quantity of bleeding)? 5. Problems with your fertility (e.g. subfertility or sterility)? 6. Change in sensations of your genitals (e.g. uncomfortable feeling or hot flushes)?   **Functions of movement and mobility**   1. Problems with your joints (e.g. ease of movement or dislocation)? 2. Muscle weakness or (semi-)paralysis (e.g. weakness or paralysis on one side of the body)? 3. Tension in muscles? 4. Problems with control and coordination of voluntary movements? 5. Involuntary movements (e.g. tremor or tic)? 6. Distorted walking pattern (e.g. swaying or stiff walking pattern)? 7. Sensation of muscle stiffness?   **Functions of the skin, hair and nails**   1. Problems with your skin (e.g. change in pigmentation, sensitivity to sunlight or more sweating than usual)? 2. Unpleasant sensations of the skin (e.g. itching, burning or tingling)? 3. Problems with your hair (e.g. change in growth, color or location of hair)? 4. Problems with your nails (e.g. change in growth or quality of nails)?   **Learning and applying knowledge**   1. Problems watching (e.g. television or sporting events)? 2. Problems listening (e.g. to a radio program)? 3. Problems copying (i.e. imitating or mimicking)? 4. Problems (re)learning language? 5. Problems learning another (not native) language? 6. Problems rehearsing (e.g. repeating chords on an instrument)? 7. Problems obtaining new information (e.g. asking for facts or names of people)? 8. Problems reading? 9. Problems (re)learning to read? 10. Problems writing? 11. Problems (re)learning to write? 12. Problems (re)learning to calculate? 13. Problems learning new skills (e.g. playing a new game)? 14. Problems making decisions?   **General tasks and demands**   1. Problems carrying out a single task independently? 2. Problems doing several different things at the same time? 3. Problems functioning independently in daily life? 4. Problems handling responsibilities (e.g. taking care of children or going to work)? 5. Problems handling stress?   **Communication**   1. Problems having a conversation (e.g. starting, continuing or ending a conversation with one person or many people)? 2. Problems having a discussion (e.g. starting, continuing or ending a discussion with one person or many people)? 3. Problems using communication devices (e.g. telephone or computer)?   **Mobility**   1. Difficulty changing your body position (e.g. lying down, sitting down or standing up)? 2. Difficulty maintaining your body position (e.g. lying, sitting or standing)? 3. Difficulty transferring yourself (e.g. moving from bed to a chair)? 4. Problems lifting or carrying objects? 5. Problems using your leg and foot to handle objects (e.g. pushing or kicking an object)? 6. Problems using your hand and fingers to handle objects (e.g. picking up an object)? 7. Problems using your arm and hand to handle objects (e.g. pushing away or reaching for an object)? 8. Problems using your foot and toes to handle objects (e.g. picking up an object)? 9. Difficulty walking (e.g. short or long distances, or around obstacles)? 10. Difficulty moving around by means other than walking (e.g. climbing stairs, swimming or running)? 11. Difficulty moving around using equipment or devices (e.g. a walker or skates)? 12. Problems using private or public transportation (e.g. traveling by train or taxi)? 13. Problems driving a vehicle (e.g. a car or bicycle)? 14. Problems riding animals for transportation (e.g. a horse)?   **Self-care**   1. Difficulty washing yourself? 2. Difficulty caring for your different body parts (e.g. skin, hair or nails)? 3. Difficulty going to the bathroom? 4. Difficulty dressing yourself? 5. Difficulty eating independently? 6. Difficulty drinking independently? 7. Difficulty looking after your own health?   **Domestic life**   1. Problems acquiring a place to live? 2. Problems acquiring goods and services (e.g. shopping for food or materials)? 3. Problems preparing a meal? 4. Problems performing household tasks (e.g. cleaning or using household appliances)? 5. Problems caring for household objects (e.g. maintaining appliances or furnishings, or taking care of plants)? 6. Problems helping others (e.g. with self-care or moving around)?   **Interpersonal interactions and relationships**   1. Problems interacting with people in a socially appropriate manner (e.g. responding to a compliment or building a relationship)? 2. Problems connecting with strangers (e.g. when asking for directions or making a purchase)? 3. Problems creating and maintaining formal relationships (e.g. with an employer or service provider)? 4. Problems creating and maintaining informal social relationships (e.g. with a friend or neighbor)? 5. Problems creating and maintaining relationships with relatives (e.g. with a parent, sibling or cousin)? 6. Problems creating and maintaining romantic relationships (e.g. with a partner)?   **Major life areas**   1. Problems engaging in education (e.g. school or course)? 2. Problems engaging in activities to prepare for a job (e.g. internship or apprenticeship)? 3. Problems engaging in paid work? 4. Problems engaging in non-paid work (i.e. volunteer work)? 5. Problems doing your personal finances?   **Community, social and civic life**   1. Problems participating in community activities (e.g. clubs or organizations)? 2. Problems participating in recreation and leisure activities (e.g. sports or hobbies)? 3. Problems participating in religious or spiritual activities? 4. Problems engaging in political life and citizenship (e.g. freedom of speech or the right to vote)? | **Not**  **at all**  0  0  0  0  0  0  0  0  0  0  0  0  0  0  0  0  0  0  0  0  0  0  0  0  0  0  **Not at**  **all**  0  0  0  0  0  0  0  0  0  0  0  0  0  0  0  0  0  0  0  0  0  0  0  0  0  0  0  0  **Not at**  **all**  0  0  0  0  0  0  0  0  0  0  0  0  0  0  0  0  0  0  0  0  0  0  0  0  0  0  **Not at**  **all**  0  0  0  0  0  0  0  0  0  0  0  0  0  0  0  0  0  0  0  0  0  0  0  0  0  0  **Not at**  **all**  0  0  0  0  0  0  0  0  0  0  0  0  0  0  0  0  0  0  0  0  0  0  0  0  **Not at**  **all**  0  0  0  0  0  0  0  0  0  0  0  0  0  0  0  0  0  0 | **A little**  **bit**  1  1  1  1  1  1  1  1  1  1  1  1  1  1  1  1  1  1  1  1  1  1  1  1  1  1  **A little**  **bit**  1  1  1  1  1  1  1  1  1  1  1  1  1  1  1  1  1  1  1  1  1  1  1  1  1  1  1  1  **A little**  **bit**  1  1  1  1  1  1  1  1  1  1  1  1  1  1  1  1  1  1  1  1  1  1  1  1  1  1  **A little**  **bit**  1  1  1  1  1  1  1  1  1  1  1  1  1  1  1  1  1  1  1  1  1  1  1  1  1  1  **A little**  **bit**  1  1  1  1  1  1  1  1  1  1  1  1  1  1  1  1  1  1  1  1  1  1  1  1  **A little**  **bit**  1  1  1  1  1  1  1  1  1  1  1  1  1  1  1  1  1  1 | **Quite**  **a bit**  2  2  2  2  2  2  2  2  2  2  2  2  2  2  2  2  2  2  2  2  2  2  2  2  2  2  **Quite**  **a bit**  2  2  2  2  2  2  2  2  2  2  2  2  2  2  2  2  2  2  2  2  2  2  2  2  2  2  2  2  **Quite**  **a bit**  2  2  2  2  2  2  2  2  2  2  2  2  2  2  2  2  2  2  2  2  2  2  2  2  2  2  **Quite**  **a bit**  2  2  2  2  2  2  2  2  2  2  2  2  2  2  2  2  2  2  2  2  2  2  2  2  2  2  **Quite**  **a bit**  2  2  2  2  2  2  2  2  2  2  2  2  2  2  2  2  2  2  2  2  2  2  2  2  **A little**  **bit**  2  2  2  2  2  2  2  2  2  2  2  2  2  2  2  2  2  2 | **Very**  **much**  3  3  3  3  3  3  3  3  3  3  3  3  3  3  3  3  3  3  3  3  3  3  3  3  3  3  **Very**  **much**  3  3  3  3  3  3  3  3  3  3  3  3  3  3  3  3  3  3  3  3  3  3  3  3  3  3  3  3  **Very**  **much**  3  3  3  3  3  3  3  3  3  3  3  3  3  3  3  3  3  3  3  3  3  3  3  3  3  3  **Very**  **much**  3  3  3  3  3  3  3  3  3  3  3  3  3  3  3  3  3  3  3  3  3  3  3  3  3  3  **Very**  **much**  3  3  3  3  3  3  3  3  3  3  3  3  3  3  3  3  3  3  3  3  3  3  3  3  **Very**  **much**  3  3  3  3  3  3  3  3  3  3  3  3  3  3  3  3  3  3 |
| --- | --- | --- | --- | --- |

**Are there any symptoms or other issues related to your daily activities or quality of life that are not mentioned in the questionnaire but are relevant to your situation?**

□ No

□ Yes, please specify:

……………………………………………………………………………………………………………………………………………………………………………………………………………………………………………………………………………………………………………………………………………………………………………………………………………………………………………………………………………………………………………………………………………………………………………………………………………………………………………………………………………………………………………………

**Did anyone help you to complete the questionnaire?**

□ No, I completed the questionnaire myself

□ Yes, someone helped me completing the questionnaire

**If you had help, what kind of help did you receive?**

□ Practical help (e.g. someone read the questions to me or physically wrote down my answers)

□ Help to understand the questions

□ Supportive help (e.g. someone just sat next to me)

□ Other kind of help, please specify: ……………………………………………………………………………………………………………………………………………………………………………

……………………………………………………………………………………………………………………………………………………………………………

**If you have any other comments on the questionnaire or in general, please specify below:**

……………………………………………………………………………………………………………………………………………………………………………………………………………………………………………………………………………………………………………………………………………………………………………………………………………………………………………………………………………………………………………………………………………………………………………………………………………………………………………………………………………………………………………………

**Supplementary Table 3.** **Agreements between patients and proxies and the percentage of participants (patients, proxies, and healthcare professionals) rating the items in the survey as relevant (score 1-3) or not relevant (score 0).**

| Survey Items | Reliability test for patient – proxy dyads | | | Percentage of patients, proxies and healthcare professionals that reported an item in the survey as an issue relevant to patients | | | | | | |
| --- | --- | --- | --- | --- | --- | --- | --- | --- | --- | --- |
|  |  | | | Relevant item  (yes vs. no) | Main analysis: not relevant (score 0) versus relevant (score 1-3) | | | Sensitivity analysis: not relevant (score 0-1) versus relevant (2-3) | | |
|  | Estimand♦ | Analysis A§ | Analysis B |  | % of Patients | % of Proxy | % of HCP | % of Patients | % of Proxy | % of HCP |
| Item 1 | Kappa  Agreement n(%)  AC | 0.44  39 (65.0)  60.0 | 0.60  48 (80.0)  60.0 | No Yes AC | 46.0  54.0  113.0 | 47.9  52.1  71.0 | 3.1  96.9  65.0 | 80.4  19.6  113.0 | 85.9  14.1  71.0 | 23.1  76.9  65.0 |
| Item 2 | Kappa  Agreement n(%)  AC | 0.40  41 (68.3)  60.0 | 0.44  44 (73.3)  60.0 | No Yes AC | 55.4  44.6  112.0 | 54.9  45.1  71.0 | 0.00  100.0  64.0 | 84.8  15.2  112.0 | 88.7  11.3  71.0 | 30.3  79.7  64.0 |
| Item 3 | Kappa  Agreement n(%)  AC | 0.31  38 (63.3)  60 | 0.48  46 (76.7)  46.0 | No Yes AC | 59.3  40.7  113.0 | 54.3  45.7  70.0 | 0.00  100.0  64.0 | 91.2  8.8  113.0 | 81.4  18.6  70.0 | 14.1  85.9  64.0 |
| Item 4 | Kappa  Agreement n(%)  AC | 0.46  30 (50.0)  60.0 | 0.50  49 (81.7)  60.0 | No Yes AC | 19.4  80.4  112.0 | 25.4  47.9  71.0 | 0.00  100.0  65.0 | 65.2  34.8  112.0 | 56.3  43.7  71.0 | 16.9  83.1  65.0 |
| Item 5 | Kappa  Agreement n(%)  AC | 0.47  36 (60.0)  60.0 | 0.70  51 (85.0)  60.0 | No Yes AC | 40.2  59.8  112.0 | 52.1  47.9  71.0 | 1.5  98.5  65.0 | 73.2  26.8  112.0 | 74.6  25.4  71.0 | 33.8  66.2  65.0 |
| Item 6 | Kappa  Agreement n(%)  AC | 0.48  42 (72.4)  58.0 | 0.52  46 (79.3)  58.0 | No Yes AC | 67.9  32.1  112.0 | 71.0  29.0  69.0 | 12.3  98.5  65.0 | 84.8  15.2  112.0 | 85.8  14.5  69.0 | 63.1  36.9  65.0 |
| Item 7 | Kappa  Agreement n(%)  AC | 0.28  44 (74.6)  59.0 | 0.37  47 (79.7)  59.0 | No Yes AC | 70.3  29.7  111.0 | 80.0  20.0  70.0 | 25.0  75.0  64.0 | 92.8  7.2  111.0 | 98.6  1.4  70.0 | 84.4  15.6  64.0 |
| Item 8 | Kappa  Agreement n(%)  AC | 0.63  39 (67.2)  58.0 | 0.68  49 (84.5)  58.0 | No Yes AC | 39.6  60.4  111.0 | 37.1  62.9  70.0 | 3.1  96.9  65.0 | 74.8  25.2  111.0 | 80.0  20.0  70.0 | 27.7  72.3  65.0 |
| Item 9 | Kappa  Agreement n(%)  AC | 0.37  26 (44.8)  58.0 | 0.41  46 (79.3)  58.0 | No Yes AC | 23.4  76.6  111.0 | 20.3  72.9  70.0 | 1.6  96.9  64.0 | 66.7  33.3  111.0 | 59.4  40.6  70.0 | 12.5  87.5  64.0 |
| Item 10 | Kappa  Agreement n(%)  AC | 0.41  29 (50.0)  58.0 | 0.60  49 (84.5)  58.0 | No Yes AC | 21.6  78.4  111.0 | 27.1  72.7  70.0 | 0.00  100.0  63.0 | 64.0  36.0  111.0 | 60.0  40.0  70.0 | 11.1  88.9  63.0 |
| Item 11 | Kappa  Agreement n(%)  AC | 0.47  45 (76.3)  59.0 | 0.66  51 (86.4)  59.0 | No Yes AC | 69.8  30.4  112.0 | 75.7  24.3  70.0 | 3,2  96.8  62.0 | 91.1  8.9  112.0 | 94.3  5.7  70.0 | 37.1  62.9  62.0 |
| Item 12 | Kappa  Agreement n(%)  AC | 0.28  41(70.7)  58.0 | 0.39  45 (77.6)  58.0 | No Yes AC | 76.9  23.1  108.0 | 65.2  34.8  69.0 | 3,2  96.8  63.0 | 96.3  3.7  108.0 | 89.9  10.1  69.0 | 54.0  46.0  63.0 |
| Item 13 | Kappa  Agreement n(%)  AC | 0.50  44 (77.2)  57.0 | 0.61  47 (82.5)  57.0 | No Yes AC | 55.0  45.0  111.0 | 61.8  38.8  68.0 | 3,2  96.8  63.0 | 88.3  11.7  111.0 | 94.1  5.9  68.0 | 36.5  63.5  63.0 |
| Item 14 | Kappa  Agreement n(%)  AC | 0.36  34 (60.7)  56.0 | 0.35  38 (67.9)  56.0 | No Yes AC | 45.4  54.6  108.0 | 50.0  50.0  68.0 | 1.6  98.4  63.0 | 84.3  15.7  108.0 | 79.4  20.6  68.0 | 33.3  66.7  63.0 |
| Item 15 | Kappa  Agreement n(%)  AC | 0.31  29 (50.9)  57.0 | 0.37  39 (68.4)  57.0 | No Yes AC | 47.7  52.3  109.0 | 50.7  49.3  64.0 | 0.00  100.0  64.0 | 81.7  18.3  109.0 | 79.7  20.3  64.0 | 23.4  76.8  64.0 |
| Item 16 | Kappa  Agreement n(%)  AC | 0.44  32 (54.2)  59.0 | 0.42  42 (71.2)  59.0 | No Yes AC | 48.6  51.4  111.0 | 42.6  57.4  68.0 | 0.00  100.0  64.0 | 79.3  20.7  111.0 | 70.6  29.4  68.0 | 25.0  75.0  64.0 |
| Item 17 | Kappa  Agreement n(%)  AC | 0.51  40 (67.8)  59.0 | 0.53  46 (78.0)  59.0 | No Yes AC | 57.3  42.3  110.0 | 60.3  39.7  68.0 | 3.2  96.8  63.0 | 84.5  15.5  110.0 | 83.8  16.2  68.0 | 49.2  50.8  63.0 |
| Item 18 | Kappa  Agreement n(%)  AC | 0.47  34 (58.6)  58.0 | 0.55  45 (77.6)  58.0 | No Yes AC | 47.7  52.3  109.0 | 44.1  55.9  68.0 | 4.7  95.3  64.0 | 83.5  16.5  109.0 | 82.4  17.6  68.0 | 39.1  60.9  64.0 |
| Item 19 | Kappa  Agreement n(%)  AC | 0.24  36 (63.2)  57.0 | 0.35  42 (73.7)  57.0 | No  Yes AC | 71.8  28.2  110.0 | 58.2  41.8  67.0 | 4.7  95.3  64.0 | 95.5  4.5  110.0 | 83.6  16.4  67.0 | 42.2  57.9  64.0 |
| Item 20 | Kappa  Agreement n(%)  AC | -0.11*  46 (80.7)  57.0 | -0.11*  46 (80.7)  57.0 | No Yes AC | 87.2  12.3  109.0 | 82.1  17.9  67.0 | 3.2  93.8  63.0 | 98.2  1.8  109.0 | 97.0  3.0  67.0 | 41.3  58.7  63.0 |
| Item 21 | Kappa  Agreement n(%)  AC | 0.39  36 (62.1)  58.0 | 0.44  42 (72.4)  58.0 | No Yes AC | 53.2  46.8  111.0 | 58.7  41.8  67.0 | 0.00  100.0  64.0 | 91.0  9.0  111.0 | 89.6  10.4  67.0 | 28.1  71.9  64.0 |
| Item 22 | Kappa  Agreement n(%)  AC | 0.42  48 (81.4)  59.0 | 0.48  49 (83.1)  59.0 | No  Yes AC | 77.3  22.3  110.0 | 73.5  26.5  68.0 | 3.1  96.9  64.0 | 97.3  2.7  110.0 | 94.1  5.9  68.0 | 37.5  62.5  64.0 |
| Item 23 | Kappa  Agreement n(%)  AC | 0.48  44 (74.6)  59.0 | 0.51  47 (79.7)  59.0 | No Yes AC | 66.7  33.3  111.0 | 70.1  29.9  67.0 | 0.00  100.0  64.0 | 91.0  9.0  1111.0 | 92.5  7.5  67.0 | 21.9  78.1  64.0 |
| Item 24 | Kappa  Agreement n(%)  AC | 0.47  43 (75.4)  57.0 | 0.56  47 (82.5)  57.0 | No Yes AC | 67.3  32.7  110.0 | 76.1  23.9  67.0 | 3.2  96.8  63.0 | 90.0  10.0  110.0 | 94.0  6.0  67.0 | 47.6  52.4  63.0 |
| Item 25 | Kappa  Agreement n(%)  AC | 0.05*  42 (73.7)  57.0 | 0.05*  42 (73.7)  57.0 | No Yes AC | 75.5  25.5  110.0 | 80.6  19.4  67.0 | 14.3  85.7  63.0 | 97.3  2.7  110.0 | 98.5  1.5  67.0 | 52.4  47.6  63.0 |
| Item 26 | Kappa  Agreement n(%)  AC | 0.35  46 (80.7)  57.0 | 0.39  47 (82.5)  57.0 | No Yes AC | 73.6  26.4  114.0 | 77.6  22.4  67.0 | 10.9  89.1  64.0 | 94.5  5.5  114.0 | 95.5  4.5  67.0 | 56.3  43.8  64.0 |
| Item 27 | Kappa  Agreement n(%)  AC | 0.65  51 (83.6)  61.0 | 0.66  54 (88.5)  61.0 | No Yes AC | 71.9  28.1  114.0 | 80.3  19.7  71.0 | 3.3  96.7  60.0 | 88.6  11.4  114.0 | 93.0  7.0  71.0 | 33.3  66.7  60.0 |
| Item 28 | Kappa  Agreement n(%)  AC | 0.72  50 (83.3)  60.0 | 0.61  52 (86.7)  60.0 | No Yes AC | 78.1  21.9  114.0 | 82.9  17.1  70.0 | 0.00  100.0  60.0 | 85.1  14.9  114.0 | 88.6  11.4  70.0 | 28.3  71.7  60.0 |
| Item 29 | Kappa  Agreement n(%)  AC | 0.56  42 (70.0)  60.0 | 0.48  45 (75.0)  60.0 | No Yes AC | 58.4  41.6  113.0 | 66.2  33.8  71.0 | 20.0  80.0  60.0 | 90.3  9.7  113.0 | 91.5  8.5  71.0 | 71.7  28.3  60.0 |
| Item 30 | Kappa  Agreement n(%)  AC | 0.35  25 (48.1)  52.0 | 0.42  37 (71.2)  52.0 | No Yes AC | 60.9  39.1  110.0 | 52.3  47.7  65.0 | 18.3  81.9  60.0 | 89.1  10.9  110.0 | 80.0  20.0  65.0 | 63.3  36.7  60.0 |
| Item 31 | Kappa  Agreement n(%)  AC | 0.43  37 (66.1)  56.0 | 0.51  44 (78.6)  56.0 | No Yes AC | 62.7  37.3  110.0 | 76.8  23.2  69.0 | 26.7  73.3  60.0 | 91.8  8.2  110.0 | 87.0  13.0  69.0 | 80.0  20.0  60.0 |
| Item 32 | Kappa  Agreement n(%)  AC | 0.55  39 (66.1)  59.0 | 0.59  47 (79.7)  59.0 | No Yes AC | 50.9  49.1  110.0 | 72.5  27.5  69.0 | 1.7  98.3  60.0 | 80.0  20.0  110.0 | 85.7  14.3  69.0 | 30.0  70.0  60.0 |
| Item 33 | Kappa  Agreement n(%)  AC | 0.30  39 (68.4)  57.0 | 0.29  41 (71.9)  57.0 | No  Yes AC | 62.7  37.3  110.0 | 72.5  27.5  70.0 | 1.7  98.3  60.0 | 90.0  10.0  110.0 | 97.1  2.9  70.0 | 36.7  63.3  60.0 |
| Item 34 | Kappa  Agreement n(%)  AC | 0.34  41 (73.2)  56.0 | 0.37  43 (76.8)  56.0 | No Yes AC | 72.1  27.9  111.0 | 80.6  19.4  69 | 23.3  76.7  60.0 | 92.8  7.2  111.0 | 94.0  6.0  69.0 | 66.7  33.3  60.0 |
| Item 35 | Kappa  Agreement n(%)  AC | 0.56  49 (87.0)  57.0 | 0.64  51 (89.5)  57.0 | No Yes AC | 83.8  16.2  111.0 | 82.4  17.6  67.0 | 28.3  71.7  60.0 | 94.6  5.4  111.0 | 97.1  2.9  67.0 | 73.3  26.7  60.0 |
| Item 36 | Kappa  Agreement n(%)  AC | 0.16*  37 (66.1)  56.0 | 0.20*  40 (71.4)  56.0 | No Yes AC | 73.9  26.1  111.0 | 76.1  23.9  68.0 | 11.7  88.3  60.0 | 94.6  5.4  111.0 | 97.0  3.0  68.0 | 53.3  46.7  60.0 |
| Item 37 | Kappa  Agreement n(%)  AC | 0.54  44 (77.2)  57.0 | 0.68  50 (87.7)  57.0 | No Yes AC | 71.2  26.1  111.0 | 67.8  32.4  67.0 | 38.3  61.7  60.0 | 88.3  11.7  111.0 | 85.3  14.7  67.0 | 78.3  21.7  60.0 |
| Item 38 | Kappa  Agreement n(%)  AC | 0.12*  46 (80.7)  57.0 | 0.20*  47 (82.5)  57.0 | No  Yes AC | 82.0  18.0  111.0 | 88.2  11.8  68.0 | 45.0  55.0  60.0 | 98.2  1.8  111.0 | 95.6  4.4  68.0 | 83.3  16.7  60.0 |
| Item 39 | Kappa  Agreement n(%)  AC | 0.38  46 (82.1)  56.0 | 0.38  47 (83.9)  56.0 | No Yes AC | 81.8  18.2  110.0 | 82.4  17.6  68.0 | 45.0  55.0  60.0 | 97.3  2.7  110.0 | 95.6  4.4  68.0 | 85.0  15.0  60.0 |
| Item 40 | Kappa  Agreement n(%)  AC | 0.40  46 (82.1)  56.0 | 0.55  48 (85.7)  56.0 | No Yes AC | 74.3  25.7  109.0 | 80.9  19.1  68.0 | 18.3  81.7  60.0 | 90.8  9.2  109.0 | 95.6  4.4  68.0 | 61.7  38.3  60.0 |
| Item 41 | Kappa  Agreement n(%)  AC | 0.45  38 (67.9)  56.0 | 0.64  46 (82.1)  56.0 | No Yes AC | 47.3  52.7  110.0 | 60.3  39.7  68.0 | 5.0  95.5  60.0 | 84.5  15.5  110.0 | 82.4  17.6  68.0 | 25.0  75.0  60.0 |
| Item 42 | Kappa  Agreement n(%)  AC | 0.45  39 (72.2)  54.0 | 0.54  43 (79.6)  54.0 | No Yes AC | 66.1  33.9  109.0 | 67.2  32.8  67.0 | 11.7  88.3  60.0 | 88.1  11.9  109.0 | 86.6  13.4  67.0 | 55.0  45.0  60.0 |
| Item 43 | Kappa  Agreement n(%)  AC | 0.38  39 (72.2)  54.0 | 0.50  43 (79.6)  54.0 | No Yes AC | 67.0  33.0  109.0 | 71.6  28.4  67.0 | 18.3  81.7  60.0 | 95.4  4.6  109.0 | 94.0  6.0  67.0 | 51.7  48.3  60.0 |
| Item 44 | Kappa  Agreement n(%)  AC | 0.25  44 (72.1)  61.0 | 0.29  45 (73.8)  61.0 | No Yes AC | 75.2  24.8  113.0 | 76.1  23.9  71.0 | 25.4  74.6  59.0 | 98.2  1.8  113.0 | 95.8  4.2  71.0 | 71.2  28.8  59.0 |
| Item 45 | Kappa  Agreement n(%)  AC | 0.25  39 (65.0)  60.0 | 0.23*  42 (70.0)  60.0 | No Yes AC | 65.2  34.8  112.0 | 69.0  31.0  71.0 | 0.0  100.0  59.0 | 89.3  10.7  112.0 | 91.5  8.5  71.0 | 22.0  78.0  59.0 |
| Item 46 | Kappa  Agreement n(%)  AC | 0.18*  55 (91.7)  60.0 | 0.24*  55 (91.7)  60.0 | No Yes AC | 92.9  7.1  113.0 | 92.9  7.1  70.0 | 23.7  76.3  59.0 | 99.1  0.9  113.0 | 98.6  1.4  70.0 | 72.9  27.1  59.0 |
| Item 47 | Kappa  Agreement n(%)  AC | 0.46  53 (86.9)  61.0 | 0.41  54 (88.5)  61.0 | No Yes AC | 80.5  19.5  113.0 | 91.5  8.5  71.0 | 44.1  55.9  59.0 | 94.7  5.3  113.0 | 98.6  1.4  71.0 | 83.1  16.9  59.0 |
| Item 48 | Kappa  Agreement n(%)  AC | 0.52  55 (90.2)  61.0 | 0.63  57 (93.4)  61.0 | No Yes AC | 87.6  12.4  113.0 | 90.1  9.9  71.0 | 13.6  86.4  59.0 | 94.7  5.3  113.0 | 97.2  2.8  71.0 | 52.5  47.5  59.0 |
| Item 49 | Kappa  Agreement n(%)  AC | 0.64  49 (83.1)  59.0 | 0.56  52 (88.1)  59.0 | No Yes AC | 80.5  19.5  113.0 | 79.7  20.3  69.0 | 27.1  72.9  59.0 | 94.7  5.3  113.0 | 89.9  10.1  69.0 | 61.0  39.0  59.0 |
| Item 50 | Kappa  Agreement n(%)  AC | 0.46  53 (89.8)  59.0 | 0.63  55 (93.2)  59.0 | No Yes AC | 87.5  12.5  112.0 | 85.7  14.3  70.0 | 45.8  54.2  59.0 | 96.4  3.6  112.0 | 95.7  4.3  70.0 | 71.2  28.8  59.0 |
| Item 51 | Kappa  Agreement n(%)  AC | 0.38  56 (94.9)  59.0 | 0.38  56 (94.9)  59.0 | No Yes AC | 91.0  8.9  112.0 | 97.1  2.9  70.0 | 74.6  25.4  59.0 | 99.1  0.9  112.0 | 100.0  0.00  70.0 | 89.8  10.2  59.0 |
| Item 52 | Kappa  Agreement n(%)  AC | 0.19*  48 (88.9)  54.0 | 0.19*  48 (88.9)  54.0 | No Yes AC | 90.0  10.0  110.0 | 95.5  4.5  66.0 | 71.2  28.8  59.0 | 96.4  3.6  110.0 | 100.0  0.00  66.0 | 89.8  10.2  59.0 |
| Item 53 | Kappa  Agreement n(%)  AC | 0.32  48 (88.9)  54.0 | 0.40  49 (90.7)  54.0 | No Yes AC | 91.0  9.0  111.0 | 89.2  10.8  65.0 | 52.5  47.5  59.0 | 98.2  1.8  111.0 | 96.9  3.1  65.0 | 89.8  10.2  59.0 |
| Item 54 | Kappa  Agreement n(%)  AC | 0.48  33 (61.1)  54.0 | 0.40  38 (70.4)  54.0 | No Yes AC | 46.8  53.2  111.0 | 53.1  56.9  64.0 | 5.1  94.9  59.0 | 73.9  26.1  111.0 | 82.8  17.2  64.0 | 33.9  66.1  59.0 |
| Item 55# | Kappa  Agreement n(%)  AC | -  60 (98.4)  61.0 | -  60 (98.4)  61.0 | No Yes AC | 96.5  3.5  113.0 | 100.0  0.00  71.0 | 57.1  42.9  56.0 | 100.0  0.0  113.0 | 100.0  0.00  71.0 | 82.1  17.9  56.0 |
| Item 56 | Kappa  Agreement n(%)  AC | 1.00  61 (100.0)  61.0 | 1.00  61 (100.0)  61.0 | No Yes AC | 96.5  3.5  113.0 | 93.0  7.0.  71.0 | 50.0  50.0  56.0 | 99.1  0.9  113.0 | 98.6  1.4  71.0 | 82.1  17.9  56.0 |
| Item 57 | Kappa  Agreement n(%)  AC | 0.58  56 (93.3)  60.0 | 0.55  57 (95.0)  60.0 | No Yes AC | 93.8  6.2  113.0 | 92.9  7.1  70.0 | 39.3  60.7  56.0 | 99.1  0.9  113.0 | 97.1  2.9  70.0 | 76.8  23.2  56.0 |
| Item 58 | Kappa  Agreement n(%)  AC | 0.25  49 (81.7)  60.0 | 0.31  50 (83.3)  60.0 | No Yes AC | 75.2  24.8  113.0 | 91.4  8.6  70.0 | 32.7  67.3  55.0 | 97.3  2.7  113.0 | 95.7  4.3  70.0 | 80.0  20.0  55.0 |
| Item 59 | Kappa  Agreement n(%)  AC | 0.38  53 (91.4)  58.0 | 0.26  53 (91.4)  58.0 | No Yes AC | 91.0  9.0  111.0 | 94.3  5.7  70.0 | 19.6  80.4  56.0 | 97.3  2.7  111.0 | 98.6  1.4  70.0 | 60.7  39.3  56.0 |
| Item 60 | Kappa  Agreement n(%)  AC | -0.06*  50 (89.3)  56.0 | -0.06*  50 (89.3)  56.0 | No Yes AC | 90.9  9.1  110.0 | 92.6  66.7  68.0 | 8.9  91.1  56.0 | 97.3  2.7  110.0 | 100.0  0.0  68.0 | 44.5  55.4  56.0 |
| Item 61 | Kappa  Agreement n(%)  AC | 0.36  32 (57.1)  56.0 | 0.17*  34 (60.7)  56.0 | No Yes AC | 56.9  43.1  109.0 | 66.7  33.3  66.0 | 12.5  87.5  56.0 | 85.3  14.7  109.0 | 86.4  13.6  66.0 | 48.2  51.8  56.0 |
| Item 62 | Kappa  Agreement n(%)  AC | 0.47  39 (69.6)  56.0 | 0.55  44 (78.6)  56.0 | No Yes AC | 56.4  43.6  110.0 | 62.7  37.3  67.0 | 19.6  80.4  56.0 | 87.2  12.7  110.0 | 85.1  14.9  67.0 | 58.9  41.1  56.0 |
| Item 63 | Kappa  Agreement n(%)  AC | 0.42  46 (83.6)  55.0 | 0.56  48 (87.3)  55.0 | No Yes AC | 78.0  22.0  109.0 | 84.8  15.2  66.0 | 1.8  98.2  56.0 | 96.3  3.7  109.0 | 90.9  9.1  66.0 | 44.6  55.4  56.0 |
| Item 64 | Kappa  Agreement n(%)  AC | 0.58  45 (81.8)  55.0 | 0.54  46 (83.6)  55.0 | No Yes AC | 69.1  30.9  110.0 | 75.4  24.6  65.0 | 28.6  71.4  56.0 | 91.8  8.2  110.0 | 90.8  9.2  65.0 | 76.8  23.2  56.0 |
| Item 65 | Kappa  Agreement n(%)  AC | 0.62  49 (89.1)  55.0 | 0.57  49 (89.09)  55.0 | No  Yes AC | 84.5  15.5  110.0 | 83.1  16.9  65.0 | 44.6  55.4  56.0 | 96.4  3.6  110.0 | 98.5  1.5  65.0 | 83.9  16.1  56.0 |
| Item 66 | Kappa  Agreement n(%)  AC | 0.30  43 (81.1)  53.0 | 0.22  43 (81.1)  53.0 | No Yes AC | 76.1  23.9  109.0 | 90.6  9.4  64.0 | 18.2  81.8  55.0 | 96.3  3.7  109.0 | 96.9  3.1  64.0 | 54.5  45.5  55.0 |
| Item 67 | Kappa  Agreement n(%)  AC | 0.36  34 (63.0)  54.0 | 0.38  40 (74.1)  54.0 | No Yes AC | 68.2  31.8  110.0 | 71.9  28.1  64.0 | 49.1  50.9  55.0 | 89.1  10.9  110.0 | 87.5  12.5  64.0 | 83.6  16.4  55.0 |
| Item 68 | Kappa  Agreement n(%)  AC | 0.33  36 (60.0)  60.0 | 0.40  44 (73.3)  60.0 | No Yes AC | 62.5  37.8  112.0 | 68.9  31.4  70.0 | 7.3  92.7  55.0 | 87.5  12.5  112.0 | 87.1  12.9  70.0 | 56.4  43.6  55.0 |
| Item 69 | Kappa  Agreement n(%)  AC | 0.24  52 (86.7)  60.0 | 0.26  52 (86.7)  60.0 | No Yes AC | 82.1  17.9  112.0 | 90.0  10.0  70.0 | 29.1  70.9  55.0 | 96.4  3.6  112.0 | 98.6  1.4  70.0 | 70.9  29.1  55.0 |
| Item 70 | Kappa  Agreement n(%)  AC | 0.35  30 (50.8)  59.0 | 0.39  41 (69.5)  59.0 | No Yes AC | 57.3  42.7  110.0 | 49.3  50.7  69.0 | 10.9  89.1  55.0 | 78.2  21.8  110.0 | 66.7  33.3  69.0 | 60.0  40.0  55.0 |
| Item 71 | Kappa  Agreement n(%)  AC | -0.09*  14 (82.4)  17.0 | -0.09*  14 (82.4)  17.0 | No Yes AC | 93.0  7.0  57.0 | 87.1  12.9  31.0 | 40.0  60.0  55.0 | 96.5  3.5  57.0 | 96.8  3.2  31.0 | 80.0  20.0  55.0 |
| Item 72# | Kappa  Agreement n(%)  AC | -  31 (100.0)  31.0 | -  31 (100.0)  31.0 | No Yes AC | 93.0  7.0  86.0 | 97.7  2.3  43.0 | 27.3  72.7  55.0 | 96.5  3.5  86.0 | 97.7  2.3  43.0 | 70.9  29.1  55.0 |
| Item 73 | Kappa  Agreement n(%)  AC | 0.25  33 (84.6)  39.0 | 0.33  33 (84.6)  39.0 | No Yes AC | 88.2  11.8  93.0 | 84.6  15.4  52.0 | 49.1  50.9  55.0 | 98.9  1.1  93.0 | 92.3  7.7  52.0 | 78.2  21.8  55.0 |
| Item 74 | Kappa  Agreement n(%)  AC | 0.46  43 (71.7)  60.0 | 0.49  48 (80.0)  60.0 | No Yes AC | 67.0  33.0  112.0 | 72.9  27.1  70.0 | 25.5  74.5  55.0 | 86.6  13.4  112.0 | 87.1  12.9  70.0 | 67.3  32.7  55.0 |
| Item 75 | Kappa  Agreement n(%)  AC | 0.44  47 (78.3)  60.0 | 0.57  51 (85.0)  60.0 | No Yes AC | 73.2  26.8  112.0 | 76.8  23.2  69.0 | 1.8  98.2  55.0 | 88.4  11.6  112.0 | 89.9  10.1  69.0 | 18.2  81.8  55.0 |
| Item 76 | Kappa  Agreement n(%)  AC | 0.42  40 (67.8)  59.0 | 0.47  45 (76.3)  59.0 | No  Yes AC | 64.5  35.5  110.0 | 66.2  33.8  68.0 | 20.0  80.0  55.0 | 86.4  13.6  110.0 | 92.6  7.4  68.0 | 72.7  27.3  55.0 |
| Item 77 | Kappa  Agreement n(%)  AC | 0.54  49 (81.7)  60.0 | 0.65  52 (86.7)  60.0 | No Yes AC | 69.6  30.4  112.0 | 78.3  21.7  69.0 | 7.3  92.7  55.0 | 91.1  8.9  112.0 | 95.7  4.3  69.0 | 34.5  65.5  55.0 |
| Item 78 | Kappa  Agreement n(%)  AC | 0.32  48 (80.0)  60.0 | 0.34  48 (80.0)  60.0 | No Yes AC | 76.6  30.4  111.0 | 87.0  13.0  69.0 | 16.4  83.6  55.0 | 1.0  9.0  111.0 | 98.6  1.4  69.0 | 54.5  45.5  55.0 |
| Item 79 | Kappa  Agreement n(%)  AC | 0.53  47 (81.0)  58.0 | 0.74  52 (89.7)  58.0 | No Yes AC | 66.1  33.9  109.0 | 73.5  26.5  68.0 | 1.8  98.2  55.0 | 88.1  11.9  109.0 | 91.2  8.8  68.0 | 30.9  69.1  55.0 |
| Item 80 | Kappa  Agreement n(%)  AC | 0.56  45 (76.3)  59.0 | 0.69  52 (88.1)  59.0 | No Yes AC | 70.9  29.1  110.0 | 72.1  27.9  68.0 | 27.3  72.7  55.0 | 90.9  10.0  110.0 | 92.6  7.4  68.0 | 63.6  36.4  55.0 |
| Item 81 | Kappa  Agreement n(%)  AC | 0.37  44 (72.1)  61.0 | 0.43  48 (78.7)  61.0 | No Yes AC | 75.0  25.0  112.0 | 71.8  28.2  71.0 | 34.5  65.5  55.0 | 95.5  4.5  112.0 | 95.8  4.2  71.0 | 85.5  14.5  55.0 |
| Item 82 | Kappa  Agreement n(%)  AC | 0.29  40 (67.8)  59.0 | 0.32  43 (72.9)  59.0 | No Yes AC | 67.3  32.7  110.0 | 71.4  28.6  70.0 | 27.3  72.7  55.0 | 88.2  11.8  110.0 | 97.1  2.9  70.0 | 76.4  23.6  55.0 |
| Item 83 | Kappa  Agreement n(%)  AC | 0.39  44 (74.6)  59.0 | 0.56  48 (81.4)  59.0 | No Yes AC | 64.9  35.1  111.0 | 67.6  32.4  71.0 | 25.5  74.5  55.0 | 95.5  4.5  111.0 | 90.1  9.9  71.0 | 65.5  34.5  55.0 |
| Item 84 | Kappa  Agreement n(%)  AC | 0.40  50 (84.7)  59.0 | 0.52  51 (86.4)  59.0 | No Yes AC | 78.2  20.5  110.0 | 84.3  15.7  70.0 | 52.7  47.3  55.0 | 96.4  3.6  110.0 | 97.1  2.9  70.0 | 89.1  10.9  55.0 |
| Item 85 | Kappa  Agreement n(%)  AC | 0.64  52 (85.2)  61.0 | 0.56  53 (86.9)  61.0 | No Yes AC | 79.5  32.4  112.0 | 78.6  21.4  70.0 | 14.5  85.5  55.0 | 92.0  8.0  112.0 | 94.3  5.7  70.0 | 67.3  32.7  55.0 |
| Item 86 | Kappa  Agreement n(%)  AC | 0.40  47 (77.0)  61.0 | 0.32  48 (78.7)  61.0 | No Yes AC | 78.6  21.4  112.0 | 84.3  15.7  70.0 | 20.0  80.0  55.0 | 94.6  5.4  112.0 | 98.6  1.4  70.0 | 74.5  25.5  55.0 |
| Item 87# | Kappa  Agreement n(%)  AC | -  52 (91.2)  57.0 | -  52 (91.2)  57.0 | No Yes AC | 86.5  13.5  111.0 | 97.0  3.0  67.0 | 30.9  69.1  55.0 | 99.1  0.9  111.0 | 100.0  0.00  67.0 | 81.8  18.2  55.0 |
| Item 88 | Kappa  Agreement n(%)  AC | 0.14  46 (80.7)  57.0 | 0.20  46 (80.7)  57.0 | No Yes AC | 76.4  23.6  106.0 | 91.3  8.7  69.0 | 18.2  81.8  55.0 | 91.5  8.5  106.0 | 98.6  1.6  69.0 | 56.4  43.6  55.0 |
| Item 89 | Kappa  Agreement n(%)  AC | 0.51  43 (84.3)  51.0 | 0.56  45 (88.2)  51.0 | No Yes AC | 74.3  25.7  101.0 | 85.1  14.9  67.0 | 47.3  52.7  55.0 | 83.2  16.8  101.0 | 92.5  7.5  67.0 | 76.4  23.6  55.0 |
| Item 90 | Kappa  Agreement n(%)  AC | 0.36  43 (81.1)  53.0 | 0.30  43 (81.1)  53.0 | No Yes AC | 75.2  24.8  105.0 | 89.1  10.9  64.0 | 41.8  58.2  55.0 | 94.3  5.7  107.0 | 96.9  3.1  64.0 | 83.6  16.4  55.0 |
| Item 91 | Kappa  Agreement n(%)  AC | 0.41  42 (73.7)  57.0 | 0.47  43 (75.4)  57.0 | No Yes AC | 55.1  44.9  107.0 | 68.7  31.3  67.0 | 1.8  98.2  55.0 | 88.8  11.2  107.0 | 95.5  4.5  67.0 | 30.9  69.1  55.0 |
| Item 92 | Kappa  Agreement n(%)  AC | 0.39  41 (74.5)\  55.0 | 0.41  43 (78.2)  55.0 | No Yes AC | 70.8  29.2  106.0 | 77.3  22.7  66.0 | 3.6  96.4  55.0 | 90.6  9.4  106.0 | 90.9  9.1  66.0 | 36.4  63.6  55.0 |
| Item 93 | Kappa  Agreement n(%)  AC | 0.53  47 (94.0)  50.0 | 0.55  47 (94.0)  50.0 | No Yes AC | 85.1  14.9  101.0 | 93.8  6.2  65.0 | 18.2  81.8  55.0 | 93.1  6.9  101.0 | 98.5  1.5  65.0 | 54.5  45.5  55.0 |
| Item 94 | Kappa  Agreement n(%)  AC | 0.33  34 (63.0)  54.0 | 0.32  38 (70.4)  54.0 | No Yes AC | 62.3  37.7  106.0 | 76.9  23.1  65.0 | 1.8  98.2  55.0 | 89.6  10.4  106.0 | 90.8  9.2  65.0 | 38.2  61.8  55.0 |
| Item 95 | Kappa  Agreement n(%)  AC | 0.20  42 (84.0)  50.0 | 0.29  43 (86.0)  50.0 | No Yes AC | 83.2  16.8  101.0 | 87.7  12.3  65.0 | 16.4  83.6  55.0 | 93.1  6.9  101.0 | 96.9  3.1  65.0 | 61.8  38.2  55.0 |
| Item 96 | Kappa  Agreement n(%)  AC | 0.35  42 (82.4)  51.0 | 0.51  45 (88.2)  51.0 | No Yes AC | 80.2  19.8  101.0 | 84.4  15.6  64.0 | 12.7  87.3  55.0 | 93.1  6.9  101.0 | 95.3  4.7  64.0 | 58.2  41.8  55.0 |
| Item 97 | Kappa  Agreement n(%)  AC | 0.35  38 (10.9)  55.0 | 0.39  42 (76.4)  55.0 | No  Yes AC | 67.0  33.0  106.0 | 70.3  29.7  64.0 | 5.5  94.5  55.0 | 87.7  12.3  106.0 | 93.8  6.3  64.0 | 56.4  43.6  55.0 |
| Item 98 | Kappa  Agreement n(%)  AC | 0.58  40 (70.2)  57.0 | 0.60  46 (80.7)  57.0 | No Yes AC | 50.9  49.1  108.0 | 59.1  40.9  66.0 | 0.00  100.0  55.0 | 79.6  20.4  108.0 | 83.3  16.7  66.0 | 23.6  76.4  55.0 |
| Item 99 | Kappa  Agreement n(%)  AC | 0.31  48 (78.7)  61.0 | 0.41  50 (82.0)  61.0 | No Yes AC | 74.1  25.9  112.0 | 76.8  23.2  69.0 | 0.00  100.0  55.0 | 91.1  8.9  112.0 | 95.7  4.3  69.0 | 36.4  63.6  55.0 |
| Item 100 | Kappa  Agreement n(%)  AC | 0.29  28 (47.5)  59.0 | 0.35  40 (67.8)  59.0 | No Yes AC | 37.2  62.8  113.0 | 39.7  60.3  68.0 | 0.00  100.0  55.0 | 90.3  9.7  113.0 | 75.0  25.0  68.0 | 21.8  78.2  55.0 |
| Item 101 | Kappa  Agreement n(%)  AC | 0.47  43 (71.7)  60.0 | 0.51  48 (80.0)  60.0 | No Yes AC | 66.4  33.6  113.0 | 67.6  32.4  68.0 | 0.00  100.0  54.0 | 88.1  11.9  113.0 | 91.2  8.8  68.0 | 13.0  87.0  54.0 |
| Item 102 | Kappa  Agreement n(%)  AC | 0.39  41 (73.2)  56.0 | 0.47  46 (91.1)  56.0 | No Yes AC | 73.4  26.6  109.0 | 75.8  24.2  66.0 | 0.00  100.0  55.0 | 71.8  28.2  109.0 | 90.9  9.1  66.0 | 18.2  81.8  55.0 |
| Item 103 | Kappa  Agreement n(%)  AC | 0.42  32 (54.2)  59.0 | 0.45  43 (72.9)  59.0 | No Yes AC | 38.2  61.8  110.0 | 39.1  60.9  69.0 | 1.8  98.2  55.0 | 71.8  28.2  110.0 | 79.7  20.3  69.0 | 27.3  72.7  55.0 |
| Item 104 | Kappa  Agreement n(%)  AC | 0.19*  43 (70.5)  61.0 | 0.29  45 (73.8)  61.0 | No Yes AC | 99.7  33.3  114.0 | 71.4  28.6  70.0 | 0.00  100.0  54.0 | 85.1  14.9  114.0 | 92.9  7.1  70.0 | 38.9  61.1  54.0 |
| Item 105 | Kappa  Agreement n(%)  AC | 0.29  37 (61.7)  60.0 | 0.38  43 (71.1)  60.0 | No Yes AC | 60.2  39.8  113.0 | 65.7  34.3  70.0 | 1.9  98.1  54.0 | 81.4  18.6  113.0 | 90.0  10.0  70.0 | 33.3  66.7  54.0 |
| Item 106 | Kappa  Agreement n(%)  AC | 0.35  43 (71.1)  60.0 | 0.33  44 (73.3)  60.0 | No  Yes AC | 69.9  30.1  113.0 | 73.5  26.5  68.0 | 3.7  96.3  54.0 | 92.9  7.1  113.0 | 97.0  2.9  68.0 | 42.6  57.4  54.0 |
| Item 107 | Kappa  Agreement n(%)  AC | 0.57  44 (73.3)  60.0 | 0.51  48 (80.0)  60.0 | No Yes AC | 62.3  37.7  114.0 | 72.1  27.9  68.0 | 3.8  96.2  52.0 | 88.6  11.4  114.0 | 92.6  7.4  68.0 | 48.1  51.9  52.0 |
| Item 108 | Kappa  Agreement n(%)  AC | 0.21*  47 (77.0)  61.0 | 0.26  48 (78.7)  61.0 | No Yes AC | 76.1  23.9  113.0 | 85.5  14.5  69.0 | 13.5  86.5  52.0 | 92.0  8.0  113.0 | 97.1  2.9  69.0 | 51.9  48.1  52.0 |
| Item 109 | Kappa  Agreement n(%)  AC | 0.59  52 (88.1)  59.0 | 0.64  53 (89.8)  59.0 | No Yes AC | 76.8  23.2  113.0 | 85.1  14.9  67.0 | 1.9  98.1  52.0 | 92.9  7.1  113.0 | 94.0  6.0  67.0 | 36.5  63.5  52.0 |
| Item 110 | Kappa  Agreement n(%)  AC | 0.47  45 (77.6)  58.0 | 0.44  47 (81.0)  58.0 | No Yes AC | 69.1  30.9  110.0 | 80.9  19.4  67.0 | 7.7  92.3  52.0 | 87.3  12.7  110.0 | 88.1  11.9  67.0 | 59.6  40.4  52.0 |
| Item 111 | Kappa  Agreement n(%)  AC | 0.52  47 (83.9)  56.0 | 0.65  51 (91.1)  56.0 | No Yes AC | 73.6  26.4  110.0 | 83.3  16.7  66.0 | 9.6  904  52.0 | 90.0  10.0  110.0 | 89.4  10.4  66.0 | 61.5  38.5  52.0 |
| Item 112 | Kappa  Agreement n(%)  AC | 0.59  47 (83.9)  56.0 | 0.68  50 (89.3)  56.0 | No Yes AC | 73.4  26.6  109.0 | 80.0  20.0  65.0 | 1.9  98.1  52.0 | 89..9  10.1  109.0 | 92.3  7.7  65.0 | 40.4  59.6  52.0 |
| Item 113 | Kappa  Agreement n(%)  AC | 0.47  45 (83.3)  54.0 | 0.47  46 (85.2)  54.0 | No Yes AC | 78.9  21.1  109.0 | 84.4  15.6  64.0 | 1.9  98.1  52.0 | 93.6  6.4  109.0 | 95.3  4.7  64.0 | 44.2  55.8  52.0 |
| Item 114 | Kappa  Agreement n(%)  AC | 0.48  45 (83.3)  54.0 | 0.79  51 (94.4)  54.0 | No Yes AC | 73.6  26.4  106.0 | 83.1  16.9  65.0 | 11.3  88.7  53.0 | 88.7  11.3  106.0 | 92.3  7.7  65.0 | 60.4  39.6  53.0 |
| Item 115 | Kappa  Agreement n(%)  AC | 0.54  42 (77.8)  54.0 | 0.66  48 (88.9)  54.0 | No Yes AC | 67.6  26.4  108.0 | 75.4  24.6  65.0 | 3.8  96.2  52.0 | 85.2  14.8  108.0 | 86.2  13.8  65.0 | 26.9  73.1  52.0 |
| Item 116 | Kappa  Agreement n(%)  AC | 0.65  42 (79.2)  53.0 | 0.72  47  53.0 | No Yes AC | 63.6  36.4  107.0 | 70.3  29.7  64.0 | 3.9  96.1  51.0 | 82.2  17.8  53.0 | 89.1  10.9  64.0 | 31.4  68.6  51.0 |
| Item 117 | Kappa  Agreement n(%)  AC | 0.59  45 (88.2)  51.0 | 0.67  47 (92.2)  51.0 | No Yes AC | 81.1  18.9  106.0 | 85.9  14.1  64.0 | 5.7  94.3  53.0 | 92.5  7.5  106.0 | 95.3  4.7  64.0 | 47.2  52.8  53.0 |
| Item 118 | Kappa  Agreement n(%)  AC | 0.61  42 (82.4)  51.0 | 0.68  46 (90.2)  51.0 | No Yes AC | 80.4  19.6  107.0 | 79.0  21.0  62.0 | 9.4  90.6  53.0 | 88.8  11.2  107.0 | 91.9  8.1  62.0 | 45.3  54.7  53.0 |
| Item 119 | Kappa  Agreement n(%)  AC | 0.49  38 (73.1)  52.0 | 0.48  40 (76.9)  52.0 | No  Yes AC | 67.0  33.0  106.0 | 58.7  41.3  63.0 | 5.7  94.3  53.0 | 77.4  22.6  106.0 | 74.6  25.4  63.0 | 26.4  73.6  53.0 |
| Item 120 | Kappa  Agreement n(%)  AC | 0.22*  33 (82.5)  40.0 | 0.33  34 (85.0)  40.0 | No Yes AC | 81.1  18.9  95.0 | 92.7  7.3  55.0 | 59.6  40.4  52.0 | 85.3  14.7  95.0 | 92.7  7.3  55.0 | 80.8  19.2  52.0 |
| Item 121 | Kappa  Agreement n(%)  AC | 0.70  53 (91.4)  58.0 | 0.71  54 (93.1)  58.0 | No Yes AC | 84.2  15.8  114.0 | 89.4  10.6  66.0 | 3.8  96.2  52.0 | 96.5  3.5  114.0 | 97.0  3.0  66.0 | 42.3  57.7  52.0 |
| Item 122 | Kappa  Agreement n(%)  AC | 0.62  54 (88.5)  61.0 | 0.61  55 (90.2)  61.0 | No Yes AC | 78.9  21.1  114.0 | 89.9  10.1  69.0 | 1.9  98.1  52.0 | 95.6  4.4  114.0 | 95.7  4.3  69.0 | 50.0  50.0  52.0 |
| Item 123 | Kappa  Agreement n(%)  AC | 0.29  53 (88.3)  60.0 | 0.35  54 (90.0)  60.0 | No Yes AC | 86.7  13.3  113.0 | 94.1  5.6  68.0 | 1.9  98.1  52.0 | 95.6  4.4  113.0 | 100.0  0.0  68.0 | 46.2  53.8  52.0 |
| Item 124 | Kappa  Agreement n(%)  AC | 0.68  53 (89.8)  59.0 | 0.78  55 (93.2  59.0 | No Yes AC | 83.0  17.0  112.0 | 86.8  13.2  68.0 | 3.8  96.2  53.0 | 92.9  7.1  112.0 | 95.6  4.4  68.0 | 43.4  56.6  53.0 |
| Item 125 | Kappa  Agreement n(%)  AC | 0.66  57 (98.3)  58.0 | 0.66  57 (98.3)  58.0 | No Yes AC | 92.9  7.1  112.0 | 97.1  2.9  68.0 | 9.4  90.6  53.0 | 98.2  1.8  112.0 | 100.0  0.0  68.0 | 49.1  50.9  53.0 |
| Item 126# | Kappa  Agreement n(%)  AC | -  56 (98.2)  57.0 | -  56 (98.2)  57.0 | No Yes AC | 96.4  3.6  111.0 | 100.0  0.00  67.0 | 11.3  88.7  53.0 | 99.1  0.9  111.0 | 100.0  0.0  67.0 | 50.9  49.1  53.0 |
| Item 127 | Kappa  Agreement n(%)  AC | 0.21*  47 (83.9)  56.0 | 0.22*  47 (83.9)  56.0 | No Yes AC | 80.0  20.0  110.0 | 90.9  9.1  66.0 | 3.8  96.2  53.0 | 97.3  2.7  110.0 | 98.5  1.5  66.0 | 30.2  69.8  53.0 |
| Item 128# | Kappa  Agreement n(%)  AC | -  55 (100.0)  55.0 | -  55 (100.0)  55.0 | No Yes AC | 96.4  3.6  111.0 | 100.0  0.00  65.0 | 20.0  80.0  50.0 | 97.3  2.7  111.0 | 100.0  0.00  65.0 | 68.0  32.0  50.0 |
| Item 129 | Kappa  Agreement n(%)  AC | 0.39  47 (79.7)  59.0 | 0.40  49 (83.1)  59.0 | No Yes AC | 80.5  19.5  113.0 | 79.4  20.6  68.0 | 11.5  88.5  52.0 | 91.2  8.8  113.0 | 89.7  10.3  68.0 | 55.8  44.2  52.0 |
| Item 130 | Kappa  Agreement n(%)  AC | 0.56  48 (80.0)  60.0 | 0.50  49 (81.7)  60.0 | No Yes AC | 71.9  28.1  114.0 | 76.5  23.5  68.0 | 7.5  92.5  53.0 | 89.5  10.5  114.0 | 89.7  10.3  68.0 | 49.1  50.9  53.0 |
| Item 131 | Kappa  Agreement n(%)  AC | 0.54  47 (79.7)  59.0 | 0.65  52 (88.1)  59.0 | No Yes AC | 72.8  27.2  114.0 | 76.1  23.9  67.0 | 3.8  96.2  52.0 | 87.7  12.3  114.0 | 89.6  10.4  67.0 | 42.3  57.7  52.0 |
| Item 132 | Kappa  Agreement n(%)  AC | 0.37  45 (77.6)  58.0 | 0.45  48 (82.8)  58.0 | No Yes AC | 77.0  23.0  114.0 | 74.6  25.4  67.0 | 11.5  88.5  52.0 | 90.3  9.7  114.0 | 89.6  10.4  67.0 | 53.8  46.2  52.0 |
| Item 133 | Kappa  Agreement n(%)  AC | 0.41  41 (78.8)  52.0 | 0.42  43 (82.7)  52.0 | No Yes AC | 74.8  25.2  113.0 | 82.5  17.5  63.0 | 7.7  92.3  52.0 | 88.8  11.2  113.0 | 90.5  9.5  63.0 | 50.0  50.0  52.0 |
| Item 134 | Kappa  Agreement n(%)  AC | 0.08*  46 (78.0)  59.0 | 0.13*  47 (79.7)  59.0 | No Yes AC | 81.3  18.8  112.0 | 78.3  21.7  69.0 | 7.5  92.5  53.0 | 95.5  4.5  112.0 | 94.2  5.8  69.0 | 43.4  56.6  53.0 |
| Item 135 | Kappa  Agreement n(%)  AC | 0.48  56 (91.8)  61.0 | 0.47  57 (93.4)  61.0 | No Yes AC | 86.8  13.2  114.0 | 89.9  10.1  69.0 | 11.5  88.5  52.0 | 95.6  4.4  114.0 | 97.1  2.9  69.0 | 61.5  38.5  53.0 |
| Item 136 | Kappa  Agreement n(%)  AC | 0.41  49 (86.0)  57.0 | 0.42  49 (86.0)  57.0 | No Yes AC | 84.1  15.9  113.0 | 84.6  15.4  65.0 | 5.8  94.2  52.0 | 94.7  5.3  113.0 | 93.8  6.2  65.0 | 44.2  55.8  52.0 |
| Item 137 | Kappa  Agreement n(%)  AC | 0.12*  47 (79.7)  59.0 | 0.13*  47 (79.7)  59.0 | No Yes AC | 79.8  20.2  114.0 | 83.6  16.4  67.0 | 9.4  90.6  53.0 | 91.2  8.8  114.0 | 98.5  1.5  67.0 | 47.2  52.8  53.0 |
| Item 138 | Kappa  Agreement n(%)  AC | 0.31  49 (83.1)  59.0 | 0.22*  50 (84.7)  59.0 | No Yes AC | 79.6  20.4  113.0 | 86.6  13.4  67.0 | 11.3  88.7  53.0 | 93.8  6.2  113.0 | 97.0  3.0  67.0 | 45.3  54.7  53.0 |
| Item 139 | Kappa  Agreement n(%)  AC | 0.13*  41 (71.9)  57.0 | 0.05*  42 (73.7)  57.0 | No Yes AC | 75.9  24.1  112.0 | 78.1  21.9  67.0 | 9.6  90.4  52.0 | 92.0  8.0  112.0 | 93.8  6.3  67.0 | 38.5  61.5  52.0 |
| Item 140 | Kappa  Agreement n(%)  AC | 0.35  40 (81.6)  49.0 | 0.34  41 (83.7)  49.0 | No Yes AC | 75.5  24.5  102.0 | 83.3  16.7  66.0 | 9.8  90.2  51.0 | 87.3  12.7  102.0 | 93.9  6.1  66.0 | 41.2  58.8  51.0 |
| Item 141 | Kappa  Agreement n(%)  AC | 0.38  41 (85.4)  48.0 | 0.38  41 (85.4)  48.0 | No Yes AC | 80.2  19.8  101.0 | 87.1  12.9  62.0 | 7.8  92.2  51.0 | 89.1  10.9  101.0 | 91.9  8.1  62.0 | 31.4  68.6  51.0 |
| Item 142 | Kappa  Agreement n(%)  AC | 0.57  39 (76.5)  51.0 | 0.64  43 (84.3)  51.0 | No Yes AC | 63.2  36.8  106.0 | 66.7  33.3  63.0 | 7.7  92.3  52.0 | 79.2  20.3  106.0 | 77.8  22.2  63.0 | 19.2  80.8  52.0 |
| Item 143 | Kappa  Agreement n(%)  AC | 0.32  38 (76.0)  50.0 | 0.36  41 (82.0)  50.0 | No Yes AC | 76.5  23.2  102.0 | 81.5  18.5  65.0 | 19.2  80.8  52.0 | 85.3  14.7  102.0 | 87.7  12.3  65.0 | 57.7  42.3  52.0 |
| Item 144 | Kappa  Agreement n(%)  AC | 0.45  43 (74.1)  58.0 | 0.55  48 (82.8)  58.0 | No Yes AC | 73.0  27.0  111.0 | 68.2  31.8  66.0 | 7.8  92.2  51.0 | 91.0  9.0  111.0 | 84.8  15.2  66.0 | 33.3  66.7  51.0 |
| Item 145 | Kappa  Agreement n(%)  AC | 0.27  42 (71.2)  59.0 | 0.35  45 (76.3)  59.0 | No Yes AC | 69.1  30.9  110.0 | 73.1  26.9  67.0 | 11.8  88.2  51.0 | 86.4  13.6  110.0 | 86.6  13.4  67.0 | 49.0  51.0  51.0 |
| Item 146 | Kappa  Agreement n(%)  AC | 0.46  43 (74.1)  58.0 | 0.52  47 (81.0)  58.0 | No Yes AC | 68.5  31.5  111.0 | 66.7  33.3  69.0 | 12.8  87.2  51.0 | 85.6  14.4  111.0 | 84.1  15.9  69.0 | 51.1  48.9  51.0 |
| Item 147 | Kappa  Agreement n(%)  AC | 0.46  43 (86.0)  50.0 | 0.56  46 (92.0)  50.0 | No Yes AC | 81.7  18.3  104.0 | 89.1  10.9  64.0 | 28.0  72.0  50.0 | 92.3  7.7  104.0 | 89.1  10.9  64.0 | 74.0  26.0  50.0 |
| Item 148 | Kappa  Agreement n(%)  AC | 0.11*  49 (86.0)  57.0 | 0.18*  50 (87.7)  57.0 | No Yes AC | 88.0  12.0  108.0 | 95.5  4.5  67.0 | 34.0  66.0  50.0 | 97.2  2.8  108.0 | 97.0  3.0  67.0 | 72.0  28.0  50.0 |

*Abbreviations: Available Case (AC), Healthcare Professional (HCP).*

*Analysis A: Agreement was assessed using the 4-Likert response score ranging from 0 (not at all relevant) to 3 (very much relevant), using weighted Kappa.*

*Analysis B: Agreement assessed using no issue (score 0) versus relevant issue (scores 1-3) with standard Kappa.* ♦ Agreement n (%) is the observed difference between the patient and proxy. Kappa and weighted kappa were estimated for analyses A and B, respectively.

**Kappa or weighted Kappa is not significant at a p-value > 0.05. The darker and the lighter black highlights show which items ≥25% of the patients, ≥25% of the proxies, or ≥50% of the healthcare providers considered relevant and irrelevant, respectively.*

*#Kappa or weighted Kappa wasn’t able to be calculated because the answer between patients and proxies was constant. (Every item was yes during the test, but a few reported no during the retest.)*

**Supplementary Table 4. The percentage of content coverage of the identified PRO measures. If ≥80% of the items in the instrument were relevant, then the PRO measure was considered to have sufficient content coverage.**

| **Identified PRO measures/instruments** | | **Percentage of items that were relevant in each instrument** | | | |
| --- | --- | --- | --- | --- | --- |
|  |  | **Number of items** | **Analysis A (%)** | **Analysis B (%)** | **Analysis C (%)** |
| 10-item LASA | 10-item Linear Analog Self-Assessment | 10 | 60,0 | 60,0 | 60,0 |
| 1-item LASA | 1-item Linear Analog Self-Assessment | 1 | 100,0 | 100,0 | 100,0 |
| 5-item LASA | 5-item Linear Analog Self-Assessment | 5 | 80,0 | 80,0 | 80,0 |
| ABS | Bradburn Affect Balance Scale | 10 | 100,0 | 100,0 | 80,0 |
| ADLS | Activities of Daily Living Scale | 38 | 44,7 | 44,7 | 42,1 |
| AEP | Adverse Events Profile | 19 | 73,7 | 73,7 | 73,7 |
| A-IADL-Q | Amsterdam Instrumental Activities of Daily Living Questionnaire | 32 | 56,3 | 56,3 | 50,0 |
| ASBQ | Anterior Skull Base Questionnaire | 35 | 60,0 | 60,0 | 57,2 |
| ASDS | Acute Stress Disorder Scale | 19 | 89,5 | 89,5 | 84,2 |
| ASEX | Arizona Sexual Experiences Scale | 6 | 100,0 | 100,0 | 100,0 |
| ASI-3 | Anxiety Sensitivity Index - 3 | 19 | 100,0 | 100,0 | 50,0 |
| ASK NASAL-12 | The Anterior Skull Base Nasal Inventory 12 | 12 | 8,3 | 8,3 | 8,3 |
| BAI | Beck Anxiety Inventory | 21 | 66,7 | 61,9 | 57,1 |
| BASIQ | Brain Symptom and Impact Questionnaire | 18 | 50,0 | 50,0 | 50,0 |
| BDI-II | Beck Depression Inventory (-II) | 21 | 100,0 | 100,0 | 95,2 |
| BFI | Brief Fatigue Inventory | 10 | 100,0 | 100,0 | 70,0 |
| BICRO-39 | Brain Injury Community Rehabilitation Outcome Scale | 40 | 65,0 | 60,0 | 25,0 |
| BIS | Body Image Scale | 10 | 90,0 | 90,0 | 10,0 |
| BMIS | Brief Mood Introspection Scale | 17 | 100,0 | 100,0 | 100,0 |
| BMSC | Brain Metastases Symptom Checklist | 18 | 61,1 | 61,1 | 55,6 |
| BPI-SF | Brief Pain Inventory - Short Form | 15 | 100,0 | 100,0 | 80,0 |
| BRIEF-A | Behavior Rating Inventory of Executive Function - Adult Version | Missing | Missing | Missing | Missing |
| BSDS | Brief Sleep Disturbance Scale | 19 | 100,0 | 100,0 | 79,0 |
| CARES-SF | Cancer Rehabilitation Evaluation System - Short Form | 61 | 63,9 | 62,3 | 42,6 |
| CDSS | Cambridge Depersonalisation Scale State | 29 | 51,7 | 48,3 | 20,7 |
| CES-D-10 | Center for Epidemiologic Studies Depression Scale - 10 items | 10 | 100,0 | 100,0 | 100,0 |
| CES-D-15 | Center for Epidemiologic Studies Depression Scale - 15 | 15 | 100,0 | 100,0 | 100,0 |
| CES-D-20 | Center for Epidemiologic Studies Depression Scale - 20 items | 20 | 95,0 | 95,0 | 95,0 |
| CFQ | Cognitive Failures Questionnaire | 25 | 92,0 | 92,0 | 60,0 |
| CFS | Cancer Fatigue Scale | 15 | 100,0 | 100,0 | 100,0 |
| CIPI | Chronic Illness Problem Inventory | 63 | 57,1 | 55,6 | 44,4 |
| CIQ | Community Integration Questionnaire | 15 | 53,3 | 53,3 | 40,0 |
| CIS | Checklist Individual Strength | 20 | 85,0 | 85,0 | 80,0 |
| CSC-W59 | Cognitive Symptom Checklist - Work 59 | 59 | 79,7 | 78,0 | 50,9 |
| DADDS | Death and Dying Distress Scale | 15 | 13,3 | 13,3 | 13,3 |
| DASS-21 | Depression Anxiety Stress Scale Short Form | 21 | 81,0 | 81,0 | 81,0 |
| DASS-42 | Depression Anxiety Stress Scale | 42 | 88,1 | 88,1 | 83,3 |
| DS | Demoralization Scale | 24 | 83,3 | 83,3 | 83,3 |
| DSQ | Dexamethasone Symptom Questionnaire | 13 | 61,5 | 61,5 | 61,5 |
| DSQ-C | Dexamethasone Symptom Questionnaire - Chronic | 15 | 66,7 | 66,7 | 66,7 |
| DT (+PL) | NCCN Distress Thermometer (and Problem List) | 40 | 52,5 | 47,5 | 47,5 |
| EBIQ | European Brain Injury Questionnaire | Missing | Missing | Missing | Missing |
| EORTC QLQ-BN20 | European Organisation for Research and Treatment of Cancer - Quality of Life Questionnaire - Brain Cancer 20(+2) | 20 | 50,0 | 50,0 | 50,0 |
| EORTC QLQ-C15- PAL | European Organisation for Research and Treatment of Cancer - Quality of Life Questionnaire - Core 15 - Palliative Care | 15 | 60,0 | 60,0 | 60,0 |
| EORTC QLQ-C30 | European Organisation for Research and Treatment of Cancer - Quality of Life Questionnaire - Core 30(+3) | 30 | 65,5 | 62,1 | 55,2 |
| EORTC-QLQ-BR23 | European Organisation for Research and Treatment of Cancer - Quality of Life Questionnaire - Breast Cancer 23 | 23 | 60,9 | 56,5 | 52,2 |
| EORTC-QLQ-FA12 | European Organisation for Research and Treatment of Cancer - Quality of Life Questionnaire - Cancer Related Fatigue 12 | 12 | 91,7 | 91,7 | 91,7 |
| EORTC-QLQ-LC13 | European Organisation for Research and Treatment of Cancer - Quality of Life Questionnaire - Lung Cancer 13 | 13 | 53,9 | 46,2 | 46,2 |
| EQ-5D | EuroQoL 5 dimensions 3 levels | 6 | 50,0 | 50,0 | 33,3 |
| e-RSCL | Extended Rotterdam Symptom Checklist | 36 | 72,2 | 72,2 | 61,1 |
| ESAS-GM | Edmonton Symptom Assessment System Glioma Module | 17 | 64,7 | 64,7 | 64,7 |
| ESAS-r | Edmonton Symptom Assessment System-Revised | 10 | 70,0 | 70,0 | 70,0 |
| ESDQ | Emotional and Social Dysfunction Questionnaire | 29 | 96,6 | 96,6 | 93,1 |
| ESS | Epworth Sleepiness Scale | 8 | 100,0 | 100,0 | 25,0 |
| ESSS | Embodied Sense of Self Scale | 23 | 60,0 | 52,0 | 32,0 |
| FACIT-F | Functional Assessment of Chronic Illness Therapy-Fatigue | 13 | 84,6 | 84,6 | 69,2 |
| FACIT-SP-12 | Functional Assessment of Chronic Illness Therapy - Spiritual Well-Being - 12 | 12 | 69,2 | 69,2 | 46,2 |
| FACT-B | Functional Assessment of Cancer Therapy-Breast | 36 | 69,4 | 66,7 | 55,6 |
| FACT-Br | Functional Assessment of Cancer Therapy-Brain | 50 | 62,0 | 62,0 | 46,0 |
| FACT-cog | Functional Assessment of Cancer Therapy-Cognitive Function | 37 | 91,9 | 91,9 | 91,9 |
| FACT-G | Functional Assessment of Cancer Therapy-General | 27 | 66,7 | 63,0 | 48,2 |
| FACT-L | Functional Assessment of Cancer Therapy-Lung | 37 | 62,2 | 62,2 | 51,4 |
| FBrSI-15 | FACT Brain Symptom Index - 15 | 15 | 60,0 | 60,0 | 60,0 |
| FLIC | Functional Living Index Cancer | 22 | 68,2 | 68,2 | 50,0 |
| FLSI | FACT Lung Symptom Index | 6 | 66,7 | 66,7 | 66,7 |
| FOP-Q-SF | Fear of Progression Questionnaire Short Form | 12 | 100,0 | 100,0 | 83,3 |
| FPQLI-C | Ferrans and Powers Quality of Life Index - Cancer version | 66 | 72,7 | 72,7 | 53,0 |
| FrSBe | Frontal Systems Behavior Scale | 46 | 65,2 | 65,2 | 63,0 |
| FSQoLS | Fox Simple Quality of Life Scale | 18 | 55,6 | 55,6 | 50,0 |
| FSS | Fatigue Severity Scale | 9 | 100,0 | 100,0 | 77,8 |
| GAD-7 | Generalized Anxiety Disorder 7-item | 7 | 100,0 | 100,0 | 100,0 |
| GDS | Geriatric Depression Scale | 30 | 93,3 | 93,3 | 93,3 |
| GHQ-28 | General Health Questionnaire - 28 | 28 | 96,4 | 96,4 | 92,9 |
| GOS | Glioma Outcomes Scale | 21 | 71,4 | 71,4 | 71,4 |
| GSDS | General Sleep Disturbance Scale | Missing | Missing | Missing | Missing |
| HADS | The Hospital Anxiety and Depression scale | 14 | 100,0 | 92,9 | 85,7 |
| HDI-SF | Hamilton Depression Inventory - Short Form | 17 | 94,1 | 88,2 | 82,4 |
| HHI | Herth Hope Scale | 12 | 91,7 | 91,7 | 75,0 |
| HUI-15Q | Health Utility Index - 15 item Questionnaire | 75 | 68,0 | 54,7 | 41,3 |
| HUS | Hassles and Uplifts Scale | 53 | 35,7 | 35,7 | 26,8 |
| IDS-SR | Inventory of Depressive Symptomatology - Self Report | 30 | 73,3 | 73,3 | 73,3 |
| IES-R | Impact of Events Scale-Revised | 22 | 95,5 | 90,9 | 90,9 |
| IIRS | Illness Intrusiveness Ratings | 13 | 53,9 | 38,5 | 23,1 |
| IPQ-R | Illness Perceptions Questionnaire-Revised | 74 | 29,7 | 29,7 | 29,7 |
| ISI | Insomnia Severity Index | 7 | 100,0 | 100,0 | 85,7 |
| LLFDI-F | Late Life Function and Disability Instrument-Function Component | 54 | 44,4 | 42,6 | 29,6 |
| MAT | MASCC (Multinational Association of Supportive Care in Cancer) Antiemesis Tool | 8 | 0,0 | 0,0 | 0,0 |
| MDASI-BT | MD Anderson Symptom Inventory Brain Tumor | 28 | 64,3 | 64,3 | 57,1 |
| MDASI-LC | MD Anderson Symptom Inventory Lung Cancer | 22 | 72,7 | 72,7 | 63,6 |
| MDMQ | Multidimensional Mood State Questionnaire | 30 | 100,0 | 100,0 | 100,0 |
| MFI | Multidimensional Fatigue Inventory | 20 | 95,0 | 95,0 | 95,0 |
| M-FLIE | Modified Functional Living Index-Emesis | 18 | 33,3 | 33,3 | 0,0 |
| MFSI-SF | Multidimensional Fatigue Symptom Inventory Short form | 30 | 87,7 | 87,7 | 87,7 |
| MHS | Miller Hope Scale | 40 | 80,0 | 80,0 | 80,0 |
| MOS CFS | Medical Outcomes Study Cognitive Functioning Scale | 6 | 83,3 | 83,3 | 66,7 |
| MQOLQ | McGill Quality of Life Questionnaire | 18 | 72,2 | 72,2 | 66,7 |
| MQOLS-CA2 | Multidimensional Quality of Life Scale - Cancer version 2 | 41 | 61,0 | 61,0 | 61,0 |
| MSAS | Memorial Symptom Assessment Scale | 32 | 71,9 | 71,9 | 68,8 |
| MSISQ15 | Multiple Sclerosis Intimacy and Sexuality Questionnaire | 15 | 80,0 | 80,0 | 80,0 |
| Neuro-QOL | Quality of Life in Neurological Disorders | Missing | Missing | Missing | Missing |
| Neuro-QOL Cognitive Function | Quality of Life in Neurological Disorders - Cognitive Function | 18 | 72,2 | 72,2 | 72,2 |
| Neuro-QOL Emotional & Behavioral Dyscontrol | Quality of Life in Neurological Disorders - Emotional and Behavioral Dyscontrol | 28 | 82,1 | 78,6 | 67,9 |
| NFBrSI-24 | NCCN-FACT-Brain Symptom Index - 24 | 24 | 95,3 | 95,3 | 95,3 |
| NHP | Nottingham Health Profile | 45 | 75,6 | 75,6 | 73,3 |
| NTUIS-Self | National Taiwan University Irritability Scale-Self | 18 | 83,3 | 83,3 | 55,6 |
| Osoba NV5 | Osoba Nausea and Vomiting Module | 11 | 81,8 | 54,6 | 9,1 |
| PAIS-SR | Psychosocial Adjustment to Illness Scale - Self Report | Missing | Missing | Missing | Missing |
| PANAS | Positive and Negative Affect Schedule | 20 | 100,0 | 100,0 | 100,0 |
| PASS-20 | Pain Anxiety Symptom Scale-20 items | 20 | 100,0 | 100,0 | 85,0 |
| PCL-C | PTSD Checklist-Civilian Version | 17 | 100,0 | 94,1 | 94,1 |
| PCRS | Patient Competency Rating Scale | 30 | 70,0 | 70,0 | 66,7 |
| PDEQ | Peritraumatic Dissociative Experience Questionnaire | 10 | 50,0 | 40,0 | 10,0 |
| PDI | Psychological Distress Inventory | 13 | 100,0 | 100,0 | 84,6 |
| PDS | Posttraumatic Stress Diagnostic Scale | 54 | 48,2 | 48,2 | 46,3 |
| PGIC | Patient's Global Impression of Change | 2 | 50,0 | 50,0 | 50,0 |
| PHQ-4 | Patient Health Questionnaire 4-item | 4 | 100,0 | 100,0 | 100,0 |
| PHQ-9 | Patient Health Questionnaire 9-item | 9 | 100,0 | 100,0 | 77,8 |
| PIPP | Perceived Impact of Problem Profile | 23 | 43,5 | 43,5 | 43,5 |
| POMS | Profile of Mood States | 40 | 100,0 | 100,0 | 100,0 |
| POMS-SF | Profile of Mood States - Short Form | 37 | 100,0 | 100,0 | 100,0 |
| POMS-SF-32 | Profile of Mood States - Short Form - 32 | 32 | 100,0 | 100,0 | 100,0 |
| POS | Palliative care Outcome Scale | 12 | 66,7 | 66,7 | 58,3 |
| PPQ | Patients' Perspective Questionnaire | 26 | 23,9 | 23,9 | 23,9 |
| PRMQ | Prospective-Retrospective Memory Questionnaire | 16 | 100,0 | 100,0 | 87,5 |
| PRO-CTCAE (study-specific) | Patient-Reported Outcomes version of the Common Terminology Criteria for Adverse Events | 123 | 69,9 | 69,9 | 69,9 |
| PRO-CTCAE-Anxiety | Patient-Reported Outcomes version of the Common Terminology Criteria for Adverse Events - Anxiety | 3 | 100,0 | 100,0 | 100,0 |
| PROMIS AbilityParticinSocRolesandActivities | Patient-Reported Outcomes Measurement Information System - Ability to Participate in Social Roles and Activities | 35 | 48,6 | 48,6 | 17,1 |
| PROMIS Anxiety | Patient-Reported Outcomes Measurement Information System - Anxiety | 29 | 96,6 | 96,6 | 96,6 |
| PROMIS Depression | Patient-Reported Outcomes Measurement Information System - Depression | 28 | 92,9 | 92,9 | 92,9 |
| PROMIS Depression SF | Patient-Reported Outcomes Measurement Information System - Depression - Short Form | 8 | 100,0 | 100,0 | 100,0 |
| PROMIS Fatigue | Patient-Reported Outcomes Measurement Information System - Fatigue | 54 | 100,0 | 100,0 | 83,3 |
| PROMIS Pain Intensity | Patient-Reported Outcomes Measurement Information System - Pain Intensity | 40 | 100,0 | 100,0 | 65,0 |
| PROMIS Pain Interference | Patient-Reported Outcomes Measurement Information System - Pain Interference | 35 | 100,0 | 100,0 | 68,6 |
| PROMIS Physical Function | Patient-Reported Outcomes Measurement Information System - Physical Function | 63 | 44,8 | 43,6 | 31,9 |
| PROMIS PIIP SF | Patient-Reported Outcomes Measurement Information System - Psychosocial Illness Impact-Positive - Short Form | 39 | 69,2 | 69,2 | 66,7 |
| PROMIS Sleep Disturbance | Patient-Reported Outcomes Measurement Information System - Sleep Disturbance | 27 | 100,0 | 100,0 | 100,0 |
| PROMIS Sleep-Related Impairment | Patient-Reported Outcomes Measurement Information System - Sleep-Related Impairment | 16 | 100,0 | 100,0 | 93,8 |
| PROMIS-GH | Patient-Reported Outcomes Measurement Information System - Global Health | 10 | 70,0 | 50,0 | 40,0 |
| PROMIS-Pain Behavior SF | Patient-Reported Outcomes Measurement Information System - Pain Behavior - Short Form | 7 | 100,0 | 100,0 | 71,4 |
| PSQI | Pittsburgh Sleep Quality Index | 24 | 95,8 | 95,8 | 66,7 |
| PSS-10 | Perceived Stress Scale 10-item | 10 | 100,0 | 100,0 | 90,0 |
| PSS-14 | Perceived Stress Scale 14-item | 14 | 100,0 | 100,0 | 100,0 |
| PTGI | Post-Traumatic Growth Inventory | 21 | 90,5 | 90,5 | 81,0 |
| PTSS-10 | Posttraumatic Symptom Scale - 10 | 10 | 90,0 | 90,0 | 90,0 |
| QOLCS | Quality of Life Cancer Survivor | 37 | 67,6 | 67,6 | 64,9 |
| QOLIE-31-P | Patient Weighted Quality of Life in Epilepsy | 45 | 86,7 | 86,7 | 84,4 |
| RSC(-ALS) | Rotterdam Symptom Checklist (-Activity Level Scale) | 38 | 65,8 | 65,8 | 63,2 |
| RSE | Rosenberg Self-Esteem Scale | 10 | 100,0 | 100,0 | 100,0 |
| SAS | Zung Self-Rating Anxiety Scale | 20 | 80,0 | 80,0 | 75,0 |
| SAS-SR | Social Adjustment Scale Self-Report | 54 | 88,9 | 87,0 | 48,2 |
| SCL-90 | Symptom CheckList - 90 | 90 | 85,6 | 83,3 | 73,3 |
| SDS | Symptom Distress Scale | 11 | 54,6 | 54,6 | 54,6 |
| SF-12 | Short Form 12 Health Survey / RAND-12 / Medical Outcome study | 12 | 83,3 | 75,0 | 75,0 |
| SF-36 | Short Form 36 Health Survey / RAND-36 / Medical Outcome Study | 36 | 66,7 | 61,1 | 50,0 |
| SF-8 | Short Form 8 Health Survey / RAND-8 / Medical Outcome study | 8 | 87,5 | 87,5 | 62,5 |
| SFMPQ | Short-Form McGill Pain Questionnaire | 15 | 86,7 | 86,7 | 86,7 |
| SGPALS | Saltin & Grimby Physical Activity Level Scale | 9 | 88,9 | 66,7 | 33,3 |
| Sintenon's 15D | Sintenon's Quality of Life Questionnaire 15D | 20 | 65,0 | 50,0 | 25,0 |
| SMQ | Short-Memory Questionnaire | 14 | 100,0 | 100,0 | 85,7 |
| SNAS | Sherbrooke Neuro-oncology Assessment Scale | 30 | 63,3 | 63,3 | 53,3 |
| SNOT-22 | Sino-Nasal Outcome Test | 22 | 59,1 | 59,1 | 59,1 |
| SQ | Symptom Questionnaire | 90 | 87,8 | 87,8 | 85,6 |
| SQLI | Spitzer Quality of Life Index | 5 | 66,7 | 46,7 | 33,3 |
| SQoL | Subjective estimation of Quality of Life | 18 | 38,9 | 38,9 | 38,9 |
| SRKP | Self-Reported Karnofsky Performance |  | 42,9 | 42,9 | 42,9 |
| Study specific questionnaire #1 | Symptom relief medical marijuana | 7 | 71,4 | 71,4 | 57,1 |
| Study specific questionnaire #10 | Visual Analogue Scale pain | 1 | 100,0 | 100,0 | 100,0 |
| Study specific questionnaire #11 | Visual Analogue Scales pain, well-being, fatigue and ability to perform daily activities | 4 | 75,0 | 75,0 | 75,0 |
| Study specific questionnaire #12 | Brain Metastases Symptom Checklist | 32 | 71,9 | 59,4 | 56,3 |
| Study specific questionnaire #13 | Visual Analogue Scales Somnolence | 5 | 100,0 | 100,0 | 100,0 |
| Study specific questionnaire #14 | Burden of cognitive complaints | 36 | 88,9 | 88,9 | 58,3 |
| Study specific questionnaire #15 | Brain metastases symptom checklist | 24 | 66,7 | 66,7 | 62,5 |
| Study specific questionnaire #16 | Quality of life | 32 | 59,4 | 59,4 | 50,0 |
| Study specific questionnaire #17 | Quality of life | Missing | Missing | Missing | Missing |
| Study specific questionnaire #18 | Brain Metastasis Patient Survey | 10 | 40,0 | 40,0 | 40,0 |
| Study specific questionnaire #19 | Daily symptom assessment | 3 | 33,3 | 33,3 | 33,3 |
| Study specific questionnaire #2 | Headache | 1 | 100,0 | 100,0 | 100,0 |
| Study specific questionnaire #20 | Fear and relaxation during elective awake craniotomy | 1 | 100,0 | 100,0 | 100,0 |
| Study specific questionnaire #21 | Depression | 3 | 100,0 | 100,0 | 100,0 |
| Study specific questionnaire #22 | Cognitive and motor function | 11 | 63,6 | 63,6 | 54,6 |
| Study specific questionnaire #23 | Subjective well-being | 1 | 0,0 | 0,0 | 0,0 |
| Study specific questionnaire #24 | Quality of life | 22 | 36,4 | 36,4 | 36,4 |
| Study specific questionnaire #25 | Innsbruck Health Dimensions Questionnaire for Neurosurgical Patients (IHD-NS) | 54 | 75,9 | 75,9 | 70,4 |
| Study specific questionnaire #26 | Quality of Life Measurement: the Toronto Instrument | 29 | 55,2 | 55,2 | 55,2 |
| Study specific questionnaire #27 | Quality of life Questionnaire | 4 | 78,7 | 78,7 | 76,6 |
| Study specific questionnaire #28 | Somnolence daily diary | 11 | 90,9 | 90,9 | 90,9 |
| Study specific questionnaire #29 | PRESTON Profile | 35 | 65,7 | 65,7 | 51,4 |
| Study specific questionnaire #3 | Surgery-related discomfort and anxiety | 15 | 60,0 | 60,0 | 60,0 |
| Study specific questionnaire #30 | Quality of life Questionnaire | 54 | 81,5 | 81,5 | 81,5 |
| Study specific questionnaire #31 | Physical and cognitive symptoms | 39 | 94,9 | 94,9 | 92,3 |
| Study specific questionnaire #32 | Well-being L.A.S.A. | 364 | 63,5 | 61,8 | 52,2 |
| Study specific questionnaire #33 | Prediagnostic brain tumor related symptoms | 29 | 58,6 | 58,6 | 44,8 |
| Study specific questionnaire #34 | Device-Specific Questionnaire | 93 | 57,0 | 54,8 | 52,7 |
| Study specific questionnaire #35 | Impact of treatment on quality of life | 4 | 25,0 | 25,0 | 25,0 |
| Study specific questionnaire #36 | Leisure Time Physical Activity Level Questionnaire | 63 | 74,6 | 71,4 | 66,7 |
| Study specific questionnaire #37 | Visual Analogue Scale Cosmetic Outcome | 1 | 100,0 | 100,0 | 0,0 |
| Study specific questionnaire #38 | Symptoms after radiotherapy | 15 | 60,0 | 60,0 | 60,0 |
| Study specific questionnaire #4 | Subjective cognitive complaints | 31 | 87,1 | 87,1 | 80,7 |
| Study specific questionnaire #5 | Anxiety and mood | 1 | 100,0 | 100,0 | 100,0 |
| Study specific questionnaire #6 | ICF-checklist activity and participation | 58 | 22,4 | 22,4 | 20,7 |
| Study specific questionnaire #7 | Impact of changes since surgery/diagnosis | 7 | 50,0 | 50,0 | 50,0 |
| Study specific questionnaire #8 | Visual Analogue Scale Pain | 99 | 60,6 | 60,6 | 53,5 |
| ST Study specific questionnaire #9 | Impact of olfactory alterations on quality of life | 1 | 0,0 | 0,0 | 0,0 |
| STAI-Y | State-Trait Anxiety Inventory Form Y | 40 | 100,0 | 100,0 | 100,0 |
| TSQ | Taste and Smell Questionnaire | 14 | 0,0 | 0,0 | 0,0 |
| USER-Participation | Utrecht Scale for Evaluation of Rehabilitation-Participation | 33 | 75,8 | 57,6 | 39,4 |
| VAMS | Visual Analog of Mood Scales | 1 | 100,0 | 100,0 | 100,0 |
| VFQ-25 | Visual Functioning Questionnaire - 25 | 25 | 48,0 | 48,0 | 4,0 |
| WHOQOL-100 | World Health Organization Quality of Life - 100 | 100 | 88,0 | 88,0 | 68,0 |
| WHOQOL-BREF | World Health Organization Quality of Life - BREF | 25 | 90,0 | 87,0 | 70,0 |
| WI-14 | Whiteley Index (hypochondriasis) - 14 | 14 | 92,9 | 92,9 | 92,9 |
| WLQ | Work Limitations Questionnaire | 43 | 86,1 | 86,1 | 69,8 |
| WPAI+CIQ:SHP | Work Productivity and Activity Impairment - Special Health Problem | 10 | 70,0 | 60,0 | 50,0 |
| WPAI-GH | Work Productivity and Activity Impairment Questionnaire-General Health | 6 | 100,0 | 100,0 | 83,3 |
| ZSDS | Zung Self-Rating Depression Scale | 20 | 95,0 | 95,0 | 95,0 |

**Analysis A:** At least one relevant aspect of an item in the instrument is relevant. E.g., ‘Doze off or fall asleep while watching TV.’ This item has one relevant aspect (falling asleep) and one irrelevant aspect (watching TV).

**Analysis B:** At least 50% of the aspects of an item in the instrument are relevant. E.g., ‘Doze off or fall asleep while watching TV.’ This item has one relevant aspect (falling asleep) and one irrelevant aspect (watching TV).

**Analysis C:** All aspects of an item in the instrument are relevant. E.g., problems with concentration and memory. This item has two aspects (concentration and memory) that are relevant.
